# Occurrence, Pollution Characteristics, Mass Load and Ecological Risk Assessment of Per- and Polyfluoroalkyl Substances in the Dianchi Basin, China

**DOI:** 10.3390/toxics14030196

**Published:** 2026-02-26

**Authors:** Hongyi Liang, Tingting Ding, Yahui Zhang, Feng Miao, Zejun Wang, Shilin Du, Jiale Cao

**Affiliations:** 1 Hebei Key Laboratory of Heavy Metal Deep-Remediation in Water and Resource Reuse, School of Environmental and Chemical Engineering, Yanshan University, Qinhuangdao 064004, China; 19932702423@163.com; 2 Environmental Analysis and Testing Laboratory, Chinese Research Academy of Environmental Sciences, Beijing 100012, China; dingtingting@craes.org.cn (T.D.); du.shilin@craes.org.cn (S.D.); 3 Bayannur Substation of the Inner Mongolia Autonomous Region Environmental Monitoring Station, Bayannur 015000, China; mf13230301882@126.com; 4 School of Environmental Science and Engineering, Suzhou University of Science and Technology, Suzhou 215009, China; wangzj9135@163.com; 5 Beijing Zhonghe Intelligent Testing Technology Service Co., Ltd., Beijing 102200, China; 17856929772@163.com

**Keywords:** Dianchi Basin, perfluoroalkyl and polyfluoroalkyl substances (PFASs), source analysis, mass loads, ecological risk

## Abstract

Per- and polyfluoroalkyl substances (PFASs) have attracted attention as emerging contaminants due to their persistence, bioaccumulation, and toxicity risks. This study investigated the characteristics, sources, mass loads, and ecological risks of 17 PFASs in surface waters and sediments from Dianchi Lake and its tributaries. During the wet season, the PFAS concentrations in the lake and river waters ranged from below the MDL (N.D.) to 11.21 ng/L and N.D. to 20.79 ng/L, respectively, while in the sediments, they were N.D. to 10.21 ng/g dry weight (dw) and N.D. to 9.63 ng/g dw. In the dry season, the lake and river water concentrations were N.D − 9.49 ng/L and N.D. − 15.67 ng/L, with those in sediments ranging from N.D. to 11.47 ng/g dw and from N.D. to 9.93 ng/g dw. Distribution coefficient analysis indicated that long-chain PFASs and sulfonic acid groups were preferentially enriched in sediments. In rivers, major sources included industrial discharges, domestic inputs, metal electroplating activities, and atmospheric deposition. In the lake, PFASs were mainly derived from mixed sources, atmospheric deposition, and riverine inflow, the latter being dominant. The PFAS loads from tributaries were estimated at 24.75 kg in the wet season and 8.79 kg in the dry season. The risk quotient values were low in waters (0.01) but ranged from 0.01 to 1 in sediments, indicating low to moderate risk, primarily from long-chain PFASs. Although ecological risk is limited, persistent inputs and contributions from tributaries highlight the necessity for continued monitoring and management. The results of this study deepen the understanding of PFAS contamination in this and other similar plateau lake basins, providing references for environmental management.

## 1. Introduction

Per- and polyfluoroalkyl substances (PFASs) are a class of synthetic organofluorine compounds that, owing to their exceptional chemical stability and surface activity, have been extensively utilized in applications such as firefighting foams, non-stick coatings, and food packaging [1]. However, this stability also results in their persistence and resistance to degradation in environmental matrices such as surface water [2], sediment [3], and the atmosphere [4]. Toxicological studies have shown that PFASs generally exhibit potential biological toxicity, including endocrine disruption, hepatotoxicity, neurotoxicity, and reproductive toxicity [5], thereby posing risks to both the ecological environment and human health. Given their persistence and biological toxicity, PFASs have now been recognized as one of the most critical emerging contaminants [6]. Long-chain PFASs (e.g., perfluorooctanoic acid (PFOA), perfluorooctanesulfonic acid (PFOS), and their salts) have been strictly regulated and gradually phased out in multiple countries [7,8]. However, studies have shown that the risk levels of short-chain alternatives (e.g., perfluorobutanoic acid) may be comparable to those of PFOA [9,10], and they also exert adverse effects on the ecological environment. As a major producer and consumer of fluorochemicals [11], China issued the “Action Plan for the Control of Emerging Contaminants” in 2022 [12], emphasizing that the identification of pollution sources is a crucial step for the effective regulation of emerging contaminants.

Various source apportionment methods have been applied to the analysis of PFAS pollution sources, including Positive Matrix Factorization (PMF) [13], Principal Component Analysis (PCA) [14], and Cluster Analysis [15]. PMF is one of the most commonly used source apportionment methods capable of quantifying potential sources and their contributions [16]. However, it primarily relies on pollutant concentration data and existing literature for analysis, often lacking in-depth consideration and interpretation of actual environmental factors. Understanding how these environmental factors influence PFASs can help predict their fate, mobility, and occurrences in water [17]. Redundancy analysis (RDA) is a method that combines PCA with correlation analysis, and it can be used to assess the contribution of environmental factors to variations in pollutant concentrations [18]. By integrating PMF with RDA and introducing environmental physicochemical factors as explanatory variables, the limitations of PMF in source contribution interpretation can be addressed, thereby providing more accurate source identification results.

Lakes are considered important sinks and transport systems for PFASs [19]. PFAS contamination has been documented in lakes located in densely populated and industrially developed regions, such as Taihu Lake [20] and Chaohu Lake [21] in China, as well as the Great Lakes in the United States [22]. PFASs in lakes primarily originate from domestic and industrial wastewater discharges, agricultural non-point source pollution, and tributary inflows [20]. Among these, studies have shown that tributary inflows are a significant source of PFASs in lakes [23]. For example, Li et al. [23] estimated the PFAS loads in Hulun Lake based on flow and pollutant concentration data, finding that the Hailar River contributed the majority (99.74%, ~15.05 kg/year) of the PFAS mass flux. In a similar study, Ma et al. [20] employed a comparable method and estimated the PFAS loads from rivers entering Taihu Lake to be approximately 1255 kg/year. In addition, the levels and spatial distribution of PFASs in lakes exhibit significant seasonal variations [24]; during the wet season, enhanced watershed runoff leads to increased external inputs, while in the dry season, extended residence time in the water body promotes the accumulation and redistribution of PFASs at the sediment–water interface [25]. Therefore, studying tributary inputs and the distribution behavior of PFASs at the water–sediment interface is of significant importance for accurately identifying the sources of PFASs in lakes.

The Yangtze River Basin is one of the seven major river basins in China and has long been subjected to various pollutants from industrial, urban wastewater and agricultural activities [26], making environmental issues a significant concern. As a typical semi-enclosed plateau lake in the upper reaches of the basin, Dianchi Lake is not only the largest plateau lake in Yunnan Province, southwest China, but also a core water body performing ecological regulation and water supply functions for the regional social–economic development of Kunming City. Its unique environmental characteristics—including a slow water exchange rate, high altitude-induced strong solar radiation, and limited water self-purification capacity—make it highly vulnerable to persistent organic pollutant (POP) accumulation and, thus, an ideal study area for exploring PFAS environmental behavior in plateau lacustrine ecosystems. For the Dianchi Lake specifically, existing investigations have confirmed the ubiquitous occurrence of PFASs in the lake’s water column, sediments, and even aquatic organisms, with short-chain PFASs (e.g., PFBA, PFBS) and long-chain PFASs (e.g., PFOA, PFOS) being the dominant congeners [27,28,29]. These studies have preliminarily revealed the basic contamination characteristics of PFASs in the lake itself, yet systematic investigations on PFASs in the basin’s inflowing river system—including their occurrence in different tributaries, spatiotemporal variation, and input contributions to the lake—remain scarce. Its catchment area encompasses 29 inflowing rivers, among which 7 discharge into the more urbanized Caohai sub-lake and 22 flow directly into the larger Waihai sub-lake, with the Tanglang River serving as the sole natural outflow channel [30]. This hydrological structure results in weak exogenous pollutant migration capacity, further exacerbating the retention and enrichment of PFASs in the lake basin. Annually, approximately 216 million cubic meters of domestic wastewater and 47.6 million cubic meters of industrial wastewater are discharged into the Dianchi Lake basin [31], along with non-point source pollution from surrounding agricultural runoff and atmospheric deposition; notably, PFASs are widely used in local textile, electroplating, food packaging, and municipal water treatment industries, and their extensive emission via multiple pathways has become a key source of PFAS contamination in the basin’s aquatic system. As a typical semi-enclosed plateau lake, the PFAS contamination status in Dianchi Lake is largely governed by exogenous inputs from its tributaries, meaning that the lack of systematic research on tributary PFASs may lead to an incomplete understanding of the entire lake basin’s pollution profile. Furthermore, few studies have been conducted to clarify the pollution sources, mass load input, and potential ecological risks of PFASs in the main tributaries of Dianchi Lake, which hinders the formulation of targeted and effective pollution control strategies for the lake basin. To address these research gaps, this study aims to (1) investigate the occurrence characteristics and spatiotemporal distribution patterns of 17 PFASs in surface water and sediments from Dianchi Lake and its major tributaries; (2) identify the sources of PFASs in the lake and its tributaries, and explore their interrelationships; (3) estimate the mass loads of PFASs transported by inflowing rivers; and (4) assess the environmental risks associated with PFASs. The findings of this study will enhance the understanding of PFAS contamination characteristics and environmental risks in Dianchi Lake and its tributaries, and provide a scientific basis for the regional environmental management and risk control of PFASs.

## 2. Materials and Methods

### 2.1. Study Area

**Figure 1:**
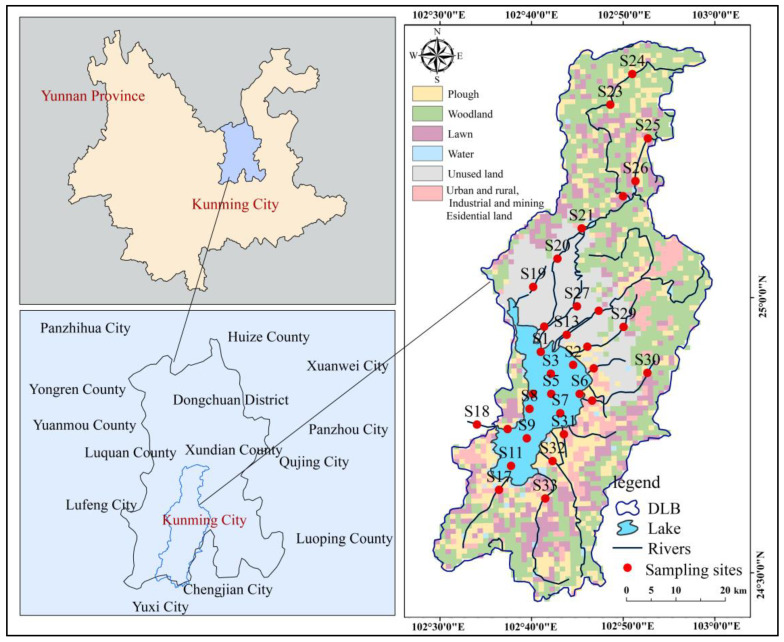
Sample sites in the river–lake system of the Dianchi Basin included 11 lake sites (S1–S11) and 22 river sites (S12–S33).

Dianchi Lake, located in Kunming, Yunnan Province, covers approximately 330 km^2^, with a total storage capacity of 1.29 billion m^3^. The Dianchi Basin encompasses the main urban areas and part of the suburban regions of Kunming City, including Wuhua, Panlong, Guandu, Xishan, Chenggong, and Jinning districts. To assess the PFAS distribution in the river–lake system, 33 sampling sites were established across the watershed (Figure 1), with lake points (S1–S11) evenly distributed in the eastern, southern, western, northern, and central regions, based on the national water quality monitoring layout. River points (S12–S33) are distributed across the major tributaries and inflows of the watershed, covering both upstream and downstream sections of medium and large reservoirs, as well as confluence areas entering the lake. Specifically, these include Xinyunliang river (S19), Panlong river (S12, S20–S24), Dianwei river (S25–S26), Haihe river (S27), Baoxiang river (S13, S28), Maliao river (S14, S29), Luolong river (S15), Laoyu river (S16, S30), Liangwang river (S31), Dahe river (S32), Dongdahe river (S33), Gucheng river (S17), and Haikou river (S18).

The rivers entering Dianchi Lake are classified into three categories based on their geographic location and pollutant characteristics: urban, agricultural, and water quality improvement-type rivers [27]. Urban-type rivers, such as Baoxiang, Maliao, Luolong, Laoyu, Xinyunliang, Haihe, Dianwei, and Panlong Rivers, flow through Kunming city and carry significant amounts of domestic and industrial wastewater. The Dahe and Dongdahe Rivers, located in intensive agricultural areas, are classified as agricultural-type rivers due to high land use intensity and frequent fertilizer and pesticide application. Liangwang and Gucheng Rivers, in the southern part of the lake, are considered water quality improvement-type estuaries due to their relatively good water quality. PFAS monitoring points were established to assess the impact of these inputs on the lake’s water environment.

### 2.2. Field Sampling

Surface water samples (2 L) were collected from a depth of 1 m using pre-cleaned glassware and stored at 4 °C for subsequent processing. Surface sediment samples were collected using stainless steel tools and stored under frozen conditions prior to processing. Sampling was conducted in July 2022 (wet season) and February 2023 (dry season). The final dataset of 66 surface water and 46 sediment samples, detailed in Table S1, reflects the omission of certain sites. Stable sediment collection was not feasible at lake site S9 and several river stations owing to field constraints such as strong hydrodynamics, hardened riverbed substrates, and anthropogenic disturbances.

### 2.3. Chemical Analyses

#### 2.3.1. Chemicals and Reagents

Seventeen types of PFAS were selected based on environmental occurrence [31], including thirteen perfluoroalkyl carboxylic acid (PFCA) groups—perfluorobutanoic acid (PFBA), perfluoropentanoic acid (PFPeA), perfluorohexanoic acid (PFHxA), perfluoroheptanoic acid (PFHpA), perfluorooctanoic acid (PFOA), perfluorononanoic acid (PFNA), perfluorodecanoic acid (PFDA), perfluoroundecanoic acid (PFUnDA), perfluorododecanoic acid (PFDoDA), perfluorotridecanoic acid (PFTrDA), perfluorotetradecanoic acid (PFTeDA), perfluorohexadecanoic acid (PFHxDA), and perfluorooctadecanoic acid (PFODA)—and four perfluoroalkyl sulfonic acid (PFSA) groups: perfluorobutane sulfonate (PFBS), perfluorohexane sulfonate (PFHxS), perfluorooctane sulfonate (PFOS), and perfluorodecane sulfonate (PFDS). Specifically, the PFCA group comprises three short-chain and ten long-chain PFASs, whereas the PFSA group consists of one short-chain and three long-chain PFASs. The chain length is defined by the number of carbon atoms in each compound (Table S2). Thirteen isotope-labeled substitute standards were used as internal standard compounds: perfluoro-n-[13C4] butanoic acid (MPFBA), perfluoro-n-[13C4] butanoic acid(MPFBA), perfluoro-n-[1,2,3,4-13C4], octanoic acid (MPFOA), perfluoro-n-[1,2,3,4,5-13C5] nonanoic acid (MPFNA), perfluoro-n-[1,2-13C2] decanoic acid (MPFDA), perfluoro-n-[1,2-13C2] undecanoic acid (MPFUnDA), perfluoro-n-[1,2-13C2] dodecanoic acid (MPFDoDA), sodium perfluoro-1-[18O2] hexanesulfonic acid (MPFHxS), and sodium perfluoro-1-[1,2,3,4-13C4] octanesulfonic acid (MPFOS). Seventeen reference compounds (98%) and nine isotopically labeled standards (98%) were purchased from Wellington (New York, NY, USA). HPLC-grade acetonitrile and methanol were obtained from Jizhi Biochemical Technology Co., Ltd. (Shanghai, China). Ammonia and ammonium acetate (CH_3_COONH_4_) were purchased from Anpuyun Experimental Supplies Co., Ltd. (Shanghai, China). The water used for HPLC analysis was sourced from Watsons Co., Ltd. (Guangzhou, China).

#### 2.3.2. Pretreatment Methods

Two liters of water were filtered through a 0.45 μm membrane, and 10 mL of a 50 ng/mL internal standard solution was added. A Waters WAX solid-phase extraction column was activated sequentially with 4 mL of 25% ammonia/methanol solution, 4 mL of methanol, and 4 mL of ultrapure water using a Waters 20-position SPE device. The water samples were passed through the column at approximately 1 drop per second to capture PFASs, followed by elution with 4 mL of 25 mmol/L ammonium acetate buffer. The column was vacuum-dried for one hour to remove residual solvents, and then eluted with methanol and 0.1% ammonia/methanol solution. The eluate was collected in a 15 mL polypropylene centrifuge tube, concentrated to 1 mL using a nitrogen blow dryer, filtered through a 0.22 μm membrane, and transferred to a 1.5 mL polypropylene liquid-phase injection vial (Agilent, Santa Clara, CA, USA) for storage at 4 °C until instrumental analysis.

The pretreatment method for PFASs in sediments was adapted from Zhang et al. [31] with modifications. The extraction procedure consisted of two steps: (a) the ultrasonic extraction of PFASs from sediments, and (b) the enrichment and purification of the extract via solid-phase extraction (SPE). A 2.0 g sediment sample, sieved through a 100-mesh sieve, was spiked with 10 μL of a 50 ng/L internal standard and 10 mL of 1% acetic acid solution. After vortex mixing, the sample was subjected to ultrasonic treatment at 40 °C for 15 min and centrifuged at 4000 rpm for 5 min, and the supernatant was collected. The sample was then extracted using a 9:1 (*v*/*v*) mixture of methanol and 1% acetic acid under ultrasonic conditions at 40 °C, with a frequency of 40 kHz, for 15 min. After centrifugation, the supernatant was collected, and the extraction was repeated twice. The extract was evaporated to 1 mL under nitrogen, filtered, and stored at 4 °C until instrumental analysis. In contrast to the pure methanol extraction commonly employed in prior research, this study utilized a mixed solvent of methanol and 1% acetic acid (90:10, *v*/*v*). The introduction of acidic conditions effectively promotes the desorption of PFASs from sediment particle surfaces, which enhances extraction efficiency while reducing matrix interferences [32].

### 2.4. Quality Assurance and Quality Control

Strict quality control and quality assurance measures were taken during sample collection and laboratory analysis. Field blanks and laboratory procedural blanks were performed for each batch of analyses. As shown in Tables S4 and S5, the main background concentrations of PFASs in the water sample field blanks were PFOA, PFNA, and PFDA. PFHxA and PFOA were also detected in the laboratory procedure blanks. No other PFAS background contamination was found in the sediment blank samples except for PFOA. The final PFAS concentrations were calculated by subtracting the corresponding blank values from the measured concentrations. In the analysis process, external standard calibration was used, with six-point calibration curves (1, 5, 10, 20, 50, and 100 ng/mL) prepared for each PFAS for quantification (*R*^2^ > 0.99). The limit of detection (LOD) and limit of quantification (LOQ) were defined as the concentrations at 3 and 10 times the signal-to-noise ratio, respectively. The LODs for target PFASs in water and sediment samples ranged from 0.01 to 0.19 ng/L and 0.02 to 0.17 ng/g, respectively. The LOQs for PFASs in water and sediment samples ranged from 0.04 to 0.61 ng/L and 0.07 to 0.56 ng/g, respectively. The recovery rates of the 17 PFAS standard compounds added to the blank samples were 79.01% to 119.03% for water and 78.41% to 124.93% for sediment samples. The recovery rates of the surrogate internal standards in water and sediment samples were as follows: MPFBA, 87.16% ± 11% and 87.51% ± 6%; MPFHxA, 92.03% ± 9% and 91.30% ± 8%; MPFOA, 91.54% ± 6% and 79.69% ± 7%; MPFNA, 79.81% ± 12% and 86.43% ± 5%; MPFDA, 87.00% ± 5% and 77.71% ± 14%; MPFUDA, 83.09% ± 14% and 84.52% ± 9%; MPFDoA, 93.75% ± 9% and 81.80% ± 6%; MPFHxS, 74.94% ± 11% and 89.22% ± 5%; MPFOS, 82.59% ± 4% and 83.74% ± 10%.

### 2.5. PFAS Source Identification

This study employed redundancy analysis (RDA) in combination with the positive matrix factorization (PMF) model to systematically identify and quantify the sources and contributions of PFAS contamination. All figures in the source apportionment section were plotted using Origin 2022 (OriginLab Corporation, Northampton, MA, USA).

#### 2.5.1. Preliminary Source Assessment

This study employed RDA to systematically investigate the influence of six key water environmental factors—suspended solids (SS), total nitrogen (TN), total phosphorus (TP), total organic carbon (TOC), chlorophyll a (Chla), and chemical oxygen demand (COD)—on the spatial distribution of PFASs. RDA was conducted using the vegan package in R 3.5.1 software (R Foundation for Statistical Computing, Vienna, Austria). RDA can simultaneously analyze the relationships between multiple response and explanatory variables, quantifying the contribution of each environmental factor to the distribution of PFASs and providing preliminary insights into the pollution sources. The measurement methods for the environmental factors are as follows: SS was determined by the gravimetric method (HJ/T 51-1999) [33], TN by potassium persulfate oxidation–UV spectrophotometry (HJ 636-2012) [34], TP by ammonium molybdate spectrophotometry (GB 11893-89) [35], TOC by high-temperature catalytic oxidation–non-dispersive infrared detection (HJ 501-2009) [36], Chla by acetone extraction–spectrophotometry (HJ 897-2017) [37], and COD by potassium dichromate oxidation (HJ 828-2017) [38]. Detailed measurements of each environmental factor are provided in Table S6.

#### 2.5.2. Positive Matrix Factorization (PMF)

The Positive Matrix Factorization model (EPA PMF 5.0, U.S. Environmental Protection Agency, Washington, DC, USA) [39] was used to identify and allocate the contributions of pollution sources. The equation can be written as follows:

(1) $$X_{ij}=\sum_{k=1}^{p}G_{ik}\times F_{kj}+E_{ij}$$

(2) $$Q=\sum_{i=1}^{n}\sum_{j=1}^{m}\frac{E_{ij}}{U_{ij}}$$

where *X_ij_* (ng/L) is the concentration of PFAS element j in sample *i*, *G_ik_* (ng/L) is the contribution of source k to sample *i*, *F_kj_* (ng/L) is the concentration of PFAS element *j* in source *k*, and *E_ij_* is the residual matrix calculated from the minimum value of the objective function *Q*, while also accounting for the uncertainty of PFAS in the samples [40]. The uncertainty is calculated as follows:

(3) $$U_{ij}=\left\{\begin{array}{c} \frac{5}{6}\times MDL,c\leq MDL\\ \sqrt{{\left(\delta \times c\right)}^{2}+{\left(MDL\right)}^{2}},\;c>MDL \end{array}\right.$$

*U_ij_* represents the uncertainty; *δ* denotes the relative error, expressed as the percentage of measurement uncertainty; *c* is the concentration (ng/L); and *MDL* refers to the method detection limit (ng/L). The PFAS concentrations, along with associated uncertainties, were incorporated to reflect the signal-to-noise ratio (S/N) of the pollutants. The selection of variables and the optimal number of factors were determined based on S/N, the convergence between *Q* robust and *Q* true, and the residual distribution of individual compounds [41,42,43].

### 2.6. Mass Load of PFASs

The estimation of pollutant mass load typically involves calculating the product of the average concentration (*C*, ng/L) of PFASs in river waters and the flow rate (*R*, m^3^/s) [44,45]. The flow data for each tributary during the two seasons were obtained through simulations using the Soil and Water Assessment Tool (SWAT) [46], with detailed results presented in Figure S1.

(4) $$M=C_{PFAS}\times R$$

The establishment of the above-mentioned formula is based on the following assumptions: (1) the adsorption of PFASs by suspended particulate matter and sediments is negligible and (2) the cumulative effects, chemical transformations, and transport losses of PFASs during the migration process are not considered [47].

### 2.7. Ecological Risk Assessment

The risk quotient (*RQ*) and total risk quotient (∑*RQ*) are commonly used for the risk characterization of exogenous substances in aquatic environments [48], and the calculation formula is shown in Equations 5 and 6. The *RQ* is defined as the ratio of the measured exposure concentration (*MEC*, ng/L) of the pollutant to the predicted no-effect concentration (*PNEC*, ng/L).

(5) $$RQ=\frac{MEC}{PNEC}$$

(6) $$\sum RQ={RQ}_{mix}=\sum_{i=1}^{n}{RQ}_{i}$$

In this study, the *PNEC* values of individual PFASs were obtained from the NORMAN Ecotoxicology Database (Table S7).

When *RQ* (∑*RQ*) <  0.01, PFASs pose no ecological risk to the target organism. When 0.01  <  *RQ* (∑*RQ*) ≤ 0.10, PFASs pose a low risk to the target organism. When 0.10  < *RQ* (∑*RQ*) ≤  1, PFASs had a moderate risk to the target organism. PFASs pose a high risk to the target organism when *RQ* (∑*RQ*) ≥  1.

### 2.8. Data Analysis

Experimental data were collated and subjected to preliminary statistical analysis using Microsoft Excel 2020 (Microsoft Corporation, Redmond, WA, USA). One-way analysis of variance (one-way ANOVA) was performed via SPSS 26.0 (IBM Corporation, Armonk, NY, USA), was adopted to analyze the seasonal variation characteristics and intergroup differences in PFAS concentrations. Redundancy analysis (RDA) was conducted with Canoco 5.0 (Microcomputer Power, Ithaca, NY, USA) to systematically explore the coupling relationships and driving effects between the physicochemical indices of water and the contents and distribution characteristics of PFASs. Statistical significance difference tests with three levels of significance set: *p* < 0.05 for significant, *p* < 0.01 for highly significant, and *p* < 0.001 for extremely significant.

## 3. Results and Discussion

### 3.1. Occurrence of PFASs in the Dianchi Basin

#### 3.1.1. PFASs in Waters

**Figure 2:**
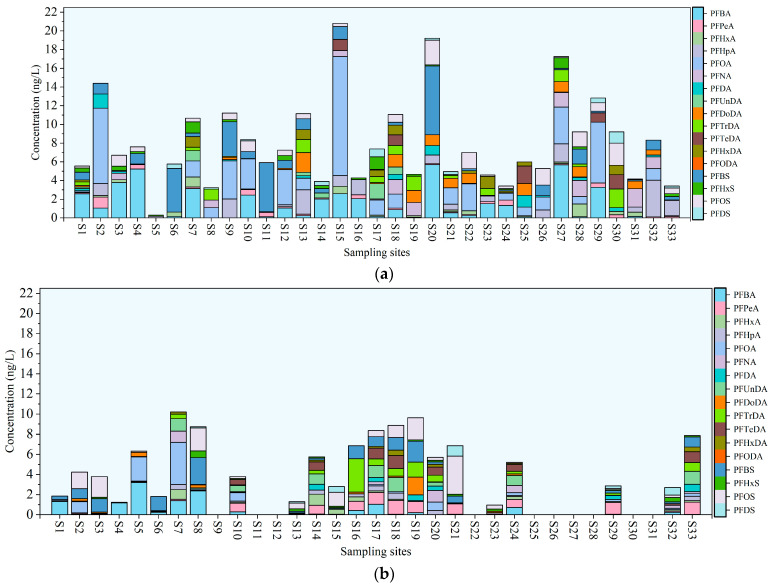
The spatial distributions of PFASs exhibit distinct pollution characteristics, wherein (**a**) shows the spatial distributions of PFASs in water samples in the wet season, and (**b**) shows the spatial distributions of PFASs in water samples in the dry season.

The concentrations of targeted PFASs at the sampling sites of the water samples are presented in Table S8 and Figure 2. The compound PFTeDA was not detected during either the wet or dry seasons. PFTeDA, a long-chain PFAS that has been increasingly restricted and phased out in recent years, was not detected in either season, likely due to its minimal environmental emissions and aqueous-phase concentrations below the LOD. During the wet season, a total of 15 PFASs were detected in the lake, and 16 in rivers during the wet season, with concentrations ranging from below the MDL (N.D.) to 11.21 ng/L (mean: 7.25) and N.D. to 20.79 ng/L (mean: 8.42 ng/L), respectively. PFOA was frequently detected, with detection rates of 54.55% (mean: 1.66 ng/L) at lake points and 63.64% (mean: 1.86 ng/L) at river points. PFOA is one of the predominant PFAS compounds in surface waters across China, with reported concentrations ranging from 0.443 to 709.44 ng/L. The concentration of PFOA detected in this study was relatively low [49]. PFOA, widely used in fluoropolymer production and surface treatment applications [50], is highly persistent and mobile in aquatic environments, which explains its frequent detection in both lakes and rivers. The detected concentrations were lower than those reported in major urban lakes such as Chaohu (13.6–90.0 ng/L) [21] and Taihu (101–308 ng/L) [51] in China. The elevated PFAS concentrations in these basins are believed to be related to industrial effluents from fluorochemical plants and other human activities near certain sampling sites. This highlights the substantial impact of the local fluorochemical industry and underscores the need for further differentiation and precise identification of point sources. The coefficients of variation (CVs) of ∑PFASs in the lake and rivers ranged from 1.09 to 2.56 and 1.07 to 2.42, respectively, indicating large differences in concentrations, which may be related to the distribution of pollution sources and the self-purification capacity of water bodies [52]. As shown in the spatial distribution map in Figure 2a, the statistical concentrations of ∑PFASs in samples from lake and urban rivers were higher than those observed in agricultural and water quality improvement rivers. Urban-type rivers generally experience higher pollution loads from industrial, domestic, and commercial sources [53]. Specifically, the concentrations of PFASs in the Panlong River, Luolong River, and Hai River were significantly higher than those in other tributaries, which may be closely related to the distribution of pollution sources and the intensity of anthropogenic activities within their catchments. The areas surrounding Dianchi Lake exhibit diverse land-use types, including urban construction land, agricultural land, and aquatic ecological protection zones (Figure 1). Different land-use patterns exert significant influences on the input pathways and migration characteristics of pollutants, resulting in pronounced variations in PFAS contamination levels among tributaries. Except for PFOS, the high values of other PFASs at lake monitoring sites were mainly distributed in the central and northern regions of the lake. The northern area is affected by multiple anthropogenic sources, such as tourism-related pollution, domestic sewage discharge, and industrial effluents [54], leading to the accumulation of various PFASs in the aquatic environment. These findings further indicate that anthropogenic activities are the primary driving factors of PFAS contamination. Additionally, the high water levels and strong hydrodynamic conditions during the wet season facilitated the input of PFASs from tributaries into the lake. Previous studies have indicated that inflowing rivers are major contributors to PFAS accumulation in lakes [23,55].

In the dry season, 15 PFASs were detected in the lake, with ∑PFASs ranging from N.D. to 9.49 ng/L (mean: 5.34 ng/L), while in rivers, the ∑15 PFAS ranged from N.D. to 15.67 ng/L (mean: 6.43 ng/L). In the lake and rivers, the CVs of PFASs ranged from 1.04 to 2.23 and from 1.28 to 3.07, respectively. As shown in the spatial distribution results in Figure 2b, the statistical concentrations of PFASs at sampling sites in urban-type rivers were generally higher. Compared with the wet season, the nearshore areas of the lake exhibited lower PFAS concentrations during the present period, reflecting pronounced spatial variations in pollutant distribution patterns under different hydrological conditions. PFBS and PFBA were predominantly detected in the lake and rivers, with detection frequencies of 81.82% (mean: 1.81 ng/L) and 50.0% (mean: 2.58 ng/L), respectively. Previous studies have shown that PFAS contamination is shifting from traditional long-chain compounds toward short-chain homologues [56]. With the strict restriction or ban of long-chain PFASs (such as PFOA and PFOS), short-chain PFASs have been widely used as alternatives in products such as water-repellent textiles, food packaging materials, and firefighting foams. Short-chain PFASs exhibit higher water solubility and polarity, resulting in greater mobility and broader environmental distribution. Although their bioaccumulation potential is relatively low, previous studies have detected trace accumulation of short-chain PFASs in biological tissues such as the blood and kidneys [57]. Relevant treatment technologies and regulatory frameworks have not yet fully addressed short-chain PFASs, highlighting the urgent need for strengthened supervision and risk management.

This study further analyzed the seasonal variation characteristics of PFAS concentrations. Considering the hydrological characteristics of the Dianchi Basin, the annual runoff distribution is uneven due to the combined influence of regional physiographic and climatic conditions. In 2023, the total annual precipitation in Kunming decreased by 29.2% compared with the previous year, while surface runoff declined by 43.6%, indicating an extremely dry hydrological year [58]. The seasonal variation in PFAS concentrations within the Dianchi Basin may therefore be related to the seasonal changes in surface runoff input and the dilution effects of the water body. Zhang et al. [59] reported a similar pattern in the Minjiang River Basin, noting that high precipitation enhanced the runoff-driven transport of PFASs from terrestrial to aquatic environments, resulting in elevated mass fluxes. In summary, both natural factors—such as surface runoff and rainfall-induced flushing—and anthropogenic influences, including incomplete treatment of domestic and industrial wastewater, are potential drivers of PFAS contamination in surface environments. Moreover, the increased runoff and precipitation during the wet season exacerbate PFAS pollution in the watershed.

Overall, the ∑PFAS concentrations in the surface waters of the Dianchi Basin in this study were within the same order of magnitude (30.98–32.19 ng/L) as those reported in 2012 [29], suggesting no evident temporal accumulation trend. This may be attributed to China’s recent policy efforts in the control of emerging contaminants [60]. The concentrations of PFASs in the Dianchi Basin were comparable to those reported for other urban lakes in China, such as Chaohu Lake (13.6–90.0 ng/L) [21] and Taihu Lake (101–308 ng/L) [51]. In these regions, PFAS contamination is typically associated with industrial discharges from fluorochemical enterprises located near sampling sites, indicating that fluorochemical industrial activities play a critical role in determining PFAS levels in aquatic environments. Furthermore, these findings highlight the importance of identifying and distinguishing point-source pollution to achieve more accurate source control and targeted remediation of PFAS contamination. International studies have shown that urban watersheds located in low- and middle-income countries, which lack sanitation infrastructure, are potential recipients of waste containing PFAS [61]. For example, Asan Lake, one of the largest reclaimed estuarine lakes in South Korea, has been undergoing continuous construction of large industrial complexes in its upstream area, with PFAS concentrations in the waters ranging from 17.7 to 467 ng/L [62]. In addition, the PFAS concentrations detected in Pampulha Lake, Brazil, reached the microgram-per-liter level, exceeding the results of this study [61]. Furthermore, surveys of the U.S. Great Lakes revealed that the PFAS concentrations generally exceed the U.S. Environmental Protection Agency (EPA) health advisory levels, and higher PFAS burdens are found in tributaries compared to receiving waters [63].

#### 3.1.2. PFASs in Sediment

**Figure 3:**
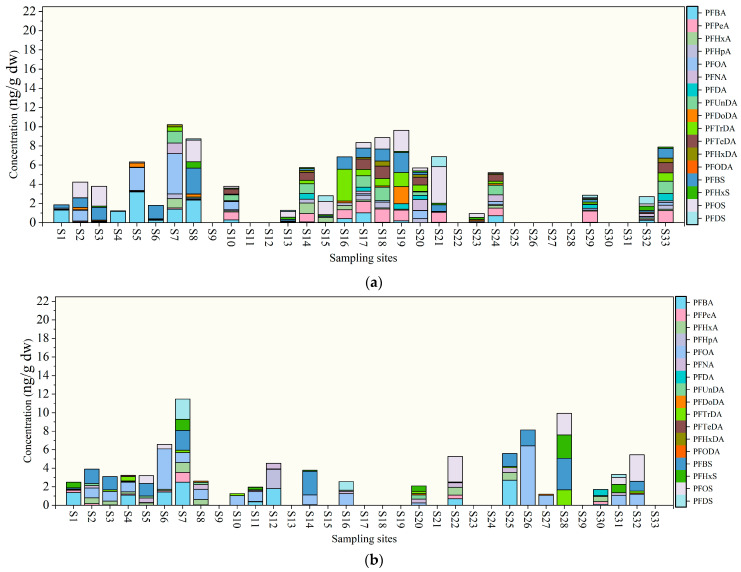
The spatial distributions of PFASs exhibit distinct pollution characteristics, wherein (**a**) shows the spatial distributions of PFASs in sediment samples in the wet season, and (**b**) shows the spatial distributions of PFASs in sediment samples in the dry season.

In the wet season, a total of 16 PFASs were detected in sediments, with the concentration levels and spatial distribution detailed in Table S9 and Figure 3a. The concentration range of ∑PFASs in the lake and river sediments was N.D. − 10.21 ng/g dry weight (dw) (mean: 4.67 ng/g dw) and N.D. − 9.63 ng/g dw (mean: 5.41 ng/g dw), respectively. These results are consistent with previous studies on PFAS concentrations in Dianchi Lake sediment [51]. PFOA and PFBS were the dominant PFASs in lake and river sediment samples, with detection rates of 66.67% (mean: 0.98 ng/g dw) and 85.71% (mean: 0.60 ng/g dw), respectively. The concentration of ∑PFASs in lake sediments was generally higher than that in river sediments, which may be attributed to the slow water flow in the lake. The low-flow environment facilitates the settling of pollutants into the lake sediment [64]. A previous study showed that most PFASs detected in sediments are long-chain PFSAs [65]. PFOA is more frequently detected in sediments, likely due to its higher water solubility and mobility, which allows it to interact with sediments through processes such as adsorption and desorption, thereby increasing its detection probability [66]. Several PFCAs (PFPeA, PFHpA, PFUnDA, PFTeDA, and PFHxDA) showed higher concentrations in sediments from the outflowing tributaries of the lake, whereas PFSAs (PFBS, PFOS, and PFDS) were more enriched in sediments from the inflowing tributaries, particularly the Panlong River. This indicates that different types of PFAS exhibit pronounced spatial heterogeneity within the lake catchment, and the PFAS contamination status in sediments varies across different regions.

In the dry season, a total of 15 PFASs were detected in sediments, with the concentration levels and spatial distribution detailed in Table S9 and Figure 3b. The concentration range of ∑PFASs in lake and river sediments was N.D. − 11.47 ng/g dw (mean: 3.98 ng/g dw) and N.D. − 9.93 ng/g dw (mean: 4.12 ng/g dw), respectively. The concentration of ∑PFASs in river sediments in this study was higher than that reported for the Red Sea (0.57–2.60 μg/kg dw) [67], but similar to the PFAS concentration levels in Caohai [68]. PFOA was the predominant PFAS in the sediments during the dry season; the PFOA detection rates in river and lake sediments were 100% (mean: 1.19 ng/g dw) and 62% (mean: 0.95 ng/g dw), respectively. Although PFOA has been banned under the Stockholm Convention, it remains prevalent in the environment due to its past widespread industrial use. Therefore, continuous monitoring and attention to its potential risks are essential.

The concentrations of ∑PFASs in sediments observed in this study were consistent with those reported by other researchers for Dianchi Lake during the same period [62]. However, the ∑PFAS levels have increased by more than an order of magnitude compared with those in 2012 (0.95 ± 0.63 ng/g dw), indicating a clear temporal accumulation effect [69]. This trend may be attributed to the environmental persistence of PFASs and their partitioning behavior at the water–sediment interface. From the perspective of water–sediment partitioning, the distribution of PFASs between the aqueous phase and sediments is strongly influenced by their hydrophobicity, carbon chain length, functional group type, and environmental physicochemical parameters (e.g., organic carbon content). Long-chain PFASs (such as PFDS and PFTeA) exhibit higher partition coefficients (*K_d_*) and thus tend to adsorb onto suspended particles and sediments, whereas short-chain PFASs (such as PFHxA) predominantly remain in the dissolved phase and migrate more readily with water flow, warranting further investigation.

#### 3.1.3. PFAS Distribution Between Waters and Sediments

Sediments not only act as sinks for PFASs but may also serve as potential secondary sources under environmental disturbances. Calculating the sediment–water partitioning coefficient facilitates the analysis of the distribution characteristics of PFASs between sediments and aquatic phases, with the calculation method referenced from a previous study [70]. In this study, synchronous sampling and analysis were conducted at 23 sites, including 9 lake sites and 14 river sites, as shown in Tables S10 and S11.

During the wet season, the mean log *K_d_* values in the lake and rivers ranged from 1.14 to 2.55 and 1.02 to 2.90, respectively, which were slightly higher than those reported in 2012 (1.08–2.30) [71]. In the dry season, the mean log *K_d_* values in the lake and rivers ranged from 1.03 to 3.12 and 1.02 to 2.38, respectively, which were comparable to field values reported in previous studies (1.2–1.6; 2.1 ± 0.1; 2.53 ± 0.35) [71,72,73]. Previous research has indicated that sediment characteristics and water quality conditions can influence PFAS partitioning behavior under real environmental conditions. Sediment organic carbon is a key sorbent-specific parameter affecting the adsorption of PFASs in dilute aqueous systems and is therefore used to normalize partition coefficients (*Koc*) to minimize bias [74].

During the wet season, the log *Koc* values in lakes and rivers ranged from 2.02 to 3.75 and 2.02 to 4.73, respectively. Overall, the log *Koc* values increased with carbon chain length (Table S10). In addition, the log *Koc* (PFSA) values were generally higher than the log *Koc* (PFCA) values. This can be attributed to the enhanced hydrophobicity of PFASs with increasing chain length [72] and the stronger electrostatic interactions associated with sulfonate functional groups in PFSAs. Due to the ionized nature of sulfonate groups, PFSAs can form stronger electrostatic interactions with positively charged sites on sediment surfaces [66], thereby enhancing their partitioning into the organic carbon phase. In contrast, the variation in log *K_d_* was less pronounced than that in log *Koc*, reflecting differences in sorption characteristics before and after organic carbon normalization.

In the dry season, the log *Koc* values in the lake and rivers ranged from 1.69 to 4.14 and 1.97 to 3.88, respectively, which were generally higher than those in the wet season. Notably, a pattern different from that in the wet season was observed: for PFCAs, log *Koc* increased with carbon chain length (Table S11), whereas for PFSAs, log *Koc* (PFBS) > log *Koc* (PFOS). The smaller steric hindrance of the short-chain PFBS allows more effective electrostatic adsorption onto positively charged sediment surfaces [74], while the longer carbon chain of PFOS causes a shielding effect that weakens the adsorption capacity of the sulfonate group [75].

Overall, the log *Koc* values in lakes were consistently higher than those in rivers across different hydrological periods, indicating that the higher organic carbon content and longer hydraulic residence times in lakes promote PFAS adsorption and enrichment. Furthermore, the overall higher partition coefficients observed in the dry season suggest enhanced organic matter accumulation and stronger adsorption under low-flow conditions. Structurally, both the log *K_d_* and log *Koc* values for PFSAs were markedly higher than those for PFCAs, and both increased with carbon chain length. These results indicate that sulfonate-based and long-chain PFASs exhibit stronger hydrophobicity and sorption affinity, facilitating their enrichment in sediments. In general, PFAS partitioning at the sediment–water interface in the Dianchi Basin is jointly controlled by hydrological conditions, environmental type, and molecular structure, consistent with the findings of Zhao et al. [76].

### 3.2. Potential Sources of PFASs

#### 3.2.1. Preliminary Identification

**Figure 4:**
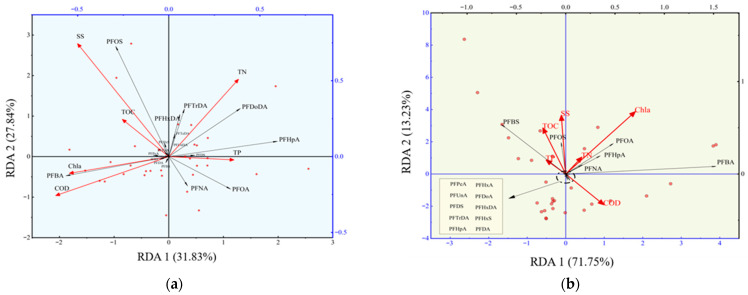
Concentration of perfluorinated compounds and water quality factors (organic matter (TOC), suspended particulate matter (SS), dissolved oxygen content (COD), total nitrogen (TN), total phosphorus (TP), and chlorophyll content (Chla)) in water bodies of rivers (**a**) and the lake (**b**).

In this study, with the exception of PFHxA, PFDA, PFoDA, PFBS, and PFHxS, the concentrations of other PFASs in sediments and water bodies exhibited a significant positive correlation (Figure S2). This suggests that the primary source of PFASs in sediments is the deposition from the overlying water column. Therefore, the concentration of PFASs in the water body was used as an indicator of their potential source. Cluster analysis of PFASs in water samples from both wet and dry periods showed consistent classification patterns (Figure S3). Based on the study by Liang et al. [77], it can be inferred that the composition of PFAS pollution sources is similar between the two water periods, and thus, the data from both periods were combined for source analysis. Further, six water quality indicators were selected as explanatory variables, with the concentration of PFASs in water bodies as the response variable, and redundancy analysis was performed. The results are presented in Figure 4.

In river samples, the cumulative explained variance of axes 1 and 2 exceeded 50%, indicating that variations in water quality factors were the primary drivers of differences in PFAS concentrations. Specifically, SS and TOC exhibited significant explanatory power for the spatial distribution differences in PFOS, PFBS, and PFDS. SS is consistent with the level of insoluble particulate matter in the water, while TOC reflects the organic matter content in the environment [27]. Existing studies have shown that inadequately treated industrial wastewater often contains paper fibers, textile fibers [78], and plastic micro-particles [79], which can lead to an increase in SS levels in water bodies [80]. It is therefore inferred that the spatial accumulation of PFOS, PFBS, and PFDS may be associated with industrial discharges. Additionally, Chla and COD showed significant explanatory power for PFBA *p* < 0.05). Domestic sewage, rich in organic substances such as carbohydrates and proteins, leads to an increase in COD levels when discharged into water bodies [81], suggesting that the accumulation of PFBA may be related to domestic sewage discharges. Furthermore, PFOA, PFNA, PFHpA, PFDoDA, PFTrDA, and PFHxA were significantly positively correlated with TN and TP. TN and TP in aquatic systems are typically derived from anthropogenic activities: agricultural fertilization activities [82], the use of phosphate-containing detergents [83], domestic sewage discharge, and inputs from certain industrial effluents [84]. They may also be influenced by atmospheric deposition and internal loading from sediments [85]. In comparison, water quality parameters showed no significant explanatory power for the spatial distribution of the remaining PFAS compounds (PFUnDA, PFHxA, PFHxS, PFPeA, PFDA, and PFTeDA) (*p* < 0.05). The spatial heterogeneity of these compounds may not be primarily governed by local water quality conditions but rather by regional or long-range transport processes. In particular, some long-chain PFASs (e.g., PFUnDA and PFTeDA) can be transported over long distances through adsorption onto atmospheric particulate matter [86]. Accordingly, we infer that their potential sources may include atmospheric deposition, wet deposition, or other long-range transport pathways [71].

The RDA results for lake samples revealed that the cumulative variance contribution of axes 1 and 2 was 84.98%, indicating that the RDA model effectively explained the relationship between water quality factors and PFAS concentrations. However, the concentration variations in most PFASs (including PFBS, PFPeA, PFHxA, PFUnA, PFDoA, PFDS, PFHxDA, PFTrDA, PFHxS, PFHpA, and PFDA) were not significantly explained by water quality factors, suggesting that these compounds may exhibit homogeneity and are primarily sourced from external pollution loads, such as atmospheric deposition or tributary inflow [23]. Among the factors that showed significant correlations, TOC and SS were positively correlated with PFOS concentrations. TOC reflects the source and accumulation of organic pollutants, while SS is consistent with the level of particulate matter in the waters [80]. On the other hand, Chla and TN were significantly correlated with the concentrations of PFASs such as PFOA and PFBA. As important indicators of the nutritional status of water bodies, the variations in Chla and TN are typically related to domestic sewage discharge and agricultural activities [83], suggesting that the sources of these PFASs may be closely linked to agricultural non-point source pollution and domestic sewage inputs.

The RDA results for both the river and lake samples indicate that water quality factors significantly influence the spatial distribution of PFASs, though there are differences in their explanatory power and source characteristics. In river samples, water quality factors such as SS, TOC, Chla, COD, TN, and TP had significant explanatory power for the concentration differences in various PFASs, suggesting that anthropogenic activities may be key drivers of the spatial distribution of PFASs in river systems. In contrast, for lake samples, the concentration variations in most PFASs were not significantly explained by water quality factors, exhibiting a strong homogeneity, with sources likely associated with external inputs, warranting further analysis.

#### 3.2.2. Source Tracing

**Figure 5:**
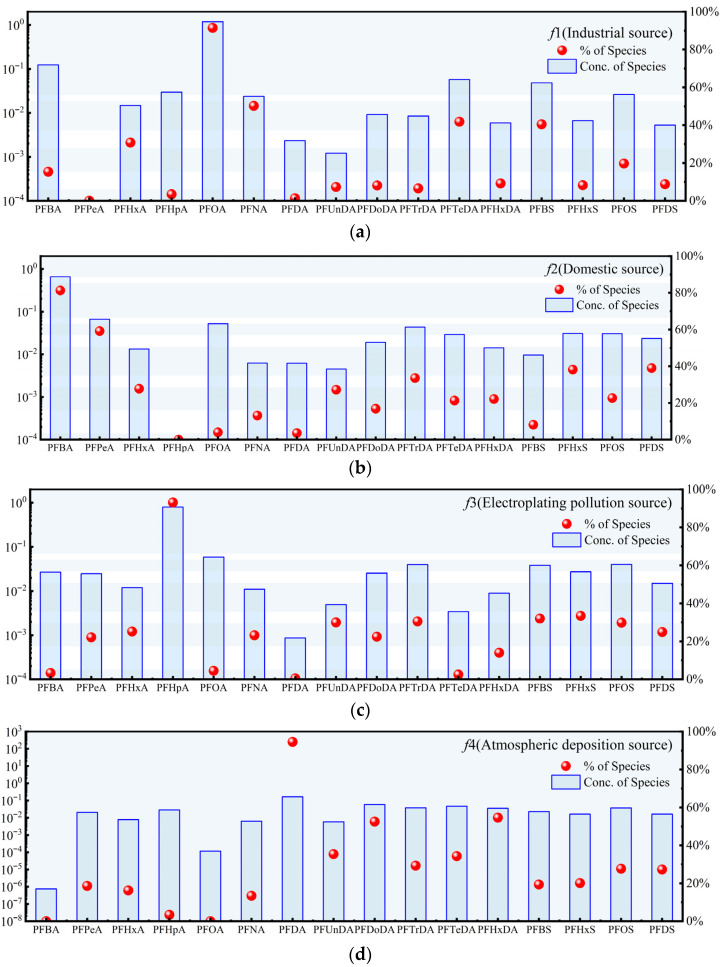
The results of positive matrix decomposition (PMF) analysis of PFASs in the river of Dianchi Basin, where (**a**) represents factor 1 (*f*1), (**b**) represents factor 2 (*f*2), (**c**) represents factor 3 (*f*3), and (**d**) represents factor 4 (*f*4).

**Figure 6:**
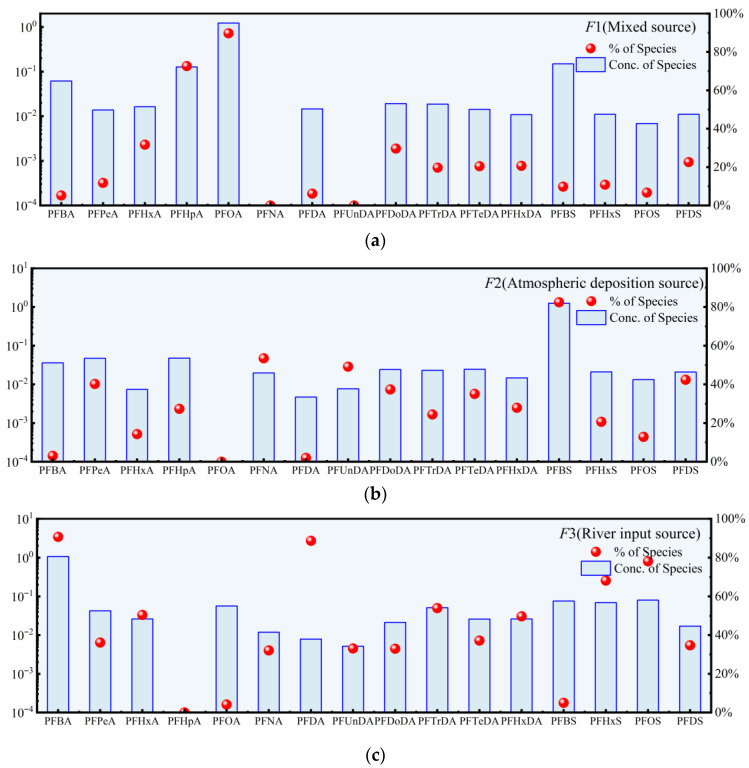
The result of positive matrix decomposition (PMF) analysis of PFASs in the lake of Dianchi Basin, where (**a**) represents factor 1 (*F*1), (**b**) represents factor 2 (*F*2), and (**c**) represents factor 3 (*F*3).

The PMF model was employed to identify the primary sources of PFASs in both river and lake samples, with the results shown in Figure 5 and Figure 6, respectively. For the river samples, the factor numbers were set to 3, 4, and 5, and the PMF model was run 20 times. When the number of factors was set to 4, the standard residuals of the elements ranged from −3 to 3, and the *Q* Robust/*Q* true ratio reached its minimum value. The model fit was satisfactory, with the fitting coefficients (*R*^2^) exceeding 0.70 (Figure S4). These findings confirm the reliability of selecting four factors [87].

Factor 1 (*f*1) is primarily driven by PFOA, PFNA, and PFTeDA, with the loadings exceeding 50%. These compounds are classified as long-chain or ultra-long-chain carboxylic acids (PFCAs). PFOA was detected at multiple sampling sites in urban-type rivers and is typically associated with the production and processing of fluoropolymer materials, particularly the synthesis and application of polytetrafluoroethylene [88]. RDA results indicated that these PFASs were correlated with TN and TP concentrations in the waters, reflecting the influence of anthropogenic activities. According to statistics, major fluorochemical enterprises within the Dianchi Basin are concentrated in the northern and western regions (Figure S5), where PFAS species and concentrations are generally higher. Moreover, the spatial hotspots of *f*1 (Figure S6) correspond closely with industrial distribution patterns, suggesting that *f*1 is primarily consistent with industrial emissions associated with the production and processing of fluorinated compounds.

Factor 2 (*f*2) exhibits high loadings on PFBA and PFPeA, both of which are short-chain PFCAs widely recognized as substitutes for traditional PFOA. Previous studies have reported that elevated levels of short-chain PFCAs substitute for PFOA in food packaging and coating materials [89]. Elevated PFBA and PFPeA concentrations are predominantly observed in urban-type rivers, and the spatial hotspots of *f*2 overlap with urban areas (Figure S6), indicating characteristics of domestic source inputs. This factor primarily originates from anthropogenic activities associated with the domestic pollution of food packaging and coating materials.

Factor 3 (*f*3) is characterized by high loadings of short-chain PFASs, including PFBS and PFDA. With the strict restrictions on PFOS under the Stockholm Convention, short-chain PFBS has been used as a substitute in the electroplating industry as a mist suppressant during chromium plating [90]. Thus, the primary source of *f*3 is associated with the use of mist suppressants in metal electroplating activities.

Factor 4 (*f*4) exhibits high loadings for various PFASs, including PFHpA, PFNA, PFUnDA, PFTrDA, PFHxS, and PFOS. PFHxS is widely used in industries such as carpet and textile production [91]; the presence of PFNA may be related to the degradation of fluoropolymer alcohols [92]; PFOS is a major byproduct in the production of aqueous film-forming foam (AFFF) in China [93]; and PFUnDA is commonly used as a textile treatment agent [92]. The sources of *f*4 exhibit a complex mixed pattern, potentially involving industrial production, product usage, and subsequent environmental transformation processes. Based on the RDA results, the spatial heterogeneity of these PFASs appears not to be governed by local water quality conditions but rather influenced by external input. Given the relatively low commercial production of PFNA, PFDA, and PFUnA, atmospheric oxidation of volatile precursor substances is considered the primary source of these compounds in the environment [29]. Therefore, the high loadings of long-chain PFASs observed in *f*4 are most likely derived from atmosphere deposition via dry and wet deposition processes.

For the lake samples, the optimal results of the PMF model were obtained through iterative analysis, with the factor number set to 3, at which point the *Q* Robust/*Q* True ratio reached the lowest convergence value. The standard residuals of the elements ranged from −3 to 3, and the *R*^2^ values were all above 0.70 (Figure S7). These findings confirm the reliability of selecting three factors. Factor 1 (F1) is characterized by high loadings of PFHpA and PFOA. As a substitute for PFOA, PFHpA is commonly used together with PFOA in food packaging and coating materials [88]. Elevated concentrations of PFHpA and PFOA in the lake are primarily distributed along the northern shoreline, near the urban area, coinciding with the high-value area of F1 (Figure S8). Therefore, F1 is likely a mixed source associated with emissions from the production and processing of fluoropolymers used in food packaging and coating materials, as well as from the use and disposal of consumer products. Xu et al. [94] similarly identified fluoropolymer production and processing as the major source of PFOA in the Dianchi Basin, consistent with the characteristics of F1 in this study. Xu et al. [94] applied PMF, PCA-MLR, and Unmix models to trace PFAS sources in the Dianchi Lake and identified, in addition to the food-packaging industry, the electroplating sector as a major emission source. Electroplating and food-packaging enterprises account for approximately 15–20% of the total industrial enterprises around the Dianchi Basin [94]. Factor (F2) exhibits high loadings on multiple PFASs, including PFPeA, PFNA, PFUnDA, PFTeDA, PFBS, and PFDS, with spatial hotspots distributed broadly, consistent with the characteristics of atmospheric deposition. Previous studies have shown that PFASs and their precursors can undergo atmospheric transport and subsequently re-enter terrestrial environments via wet and dry deposition [95]. Combined with RDA results, the spatial variability of these PFASs appears to be weakly influenced by local water quality conditions. Therefore, the pollution pattern represented by F2 is likely attributable to emissions from industrial activities such as electroplating, with PFASs entering aquatic systems primarily through atmospheric deposition. Xu et al. [94] also reported that, in addition to fluoropolymer production and electroplating industries, the third factor simulated using the PMF model exhibited low loadings for most PFASs and was classified as noise. In contrast, factor 3 (F3) in the present study showed high loadings for multiple PFASs, including PFBA, PFHxA, PFHxDA, PFHxS, and PFOS. According to the redundancy analysis and PMF spatial distribution maps (Figure S8), these PFASs were primarily influenced by external inputs, with relatively high concentrations observed in inflow rivers. The riverine input contribution of F3 is consistent with a major source of PFAS contamination in the study area.

In this study, the RDA approach was integrated with the PMF model to achieve both qualitative identification and quantitative apportionment of PFAS pollution sources. The RDA approach revealed linear relationships between PFAS concentrations and multiple water quality parameters, identifying the main potential pollution source types—anthropogenic sources and external input—thereby providing a foundation for source apportionment. Based on this, the PMF model decomposed the compound concentration matrix to quantitatively determine the compositional characteristics of each pollution source and its contribution to total PFASs.

The sources of PFASs in the lake and rivers exhibit certain similarities but also notable differences. In rivers, PFAS contamination primarily originates from industrial emissions, consumer product use, and atmospheric deposition, consistent with the source apportionment results for the Oder River [15]. Moreover, Yang et al. [96] reported that the fluorochemical industry exerts a significant influence on regional estuarine PFAS levels. Based on a structural equation model (SEM) [92,97], this study quantitatively assessed the effects of administrative division, per capita GDP, population density, and the proportion of the tertiary industry (Table S12) on ∑PFAS concentrations [98,99]. The SEM results (Figure S9) indicate that administrative division and the proportion of the tertiary industry exert positive effects on ∑PFAS concentrations, whereas per capita GDP and population density show negative effects. These findings suggest that regions with a more developed tertiary industry may experience increased PFAS inputs into rivers due to domestic emissions and consumer product usage, while suburban areas with lower GDP and population density—where wastewater treatment plants and industrial zones are typically located—may serve as indirect sources of PFAS emissions. Comparatively, PFAS pollution in lakes is more strongly influenced by atmospheric deposition and external inputs from inflowing tributaries. Previous studies have demonstrated that riverine inflow constitutes the primary source of PFASs in lakes; for instance, Li et al. [23] identified this pattern in Hulun Lake, and Sokoloya et al. [55] reported similar findings for Lake Ekoln. As another plateau lake within Kunming City, studies on Yangzonghai Lake have shown that atmospheric transport and precipitation input are the dominant sources of PFASs in its waters [100]. The findings of the present study are consistent with these results, indicating that PFASs in the lake are primarily derived from external inputs.

### 3.3. PFAS Mass Load

The influence of tributary inflow on PFAS contamination in the lake was quantified by estimating the monthly runoff (m^3^/s; Table S13) of twelve inflowing rivers traversing major and medium-sized reservoirs within the Dianchi Basin for July 2022 and February 2023, following the method proposed by [46]. Based on these estimates, the corresponding monthly PFAS mass loads entering the lake were subsequently calculated.

During the two monitored months, the total PFAS mass loads from the twelve inflowing rivers were 24.75 kg and 8.79 kg, respectively. The total load during the wet season exceeded the reported annual inflow load for Hulun Lake (15.05 kg/year) but was lower than that for Taihu Lake (1255 kg/year; [20]) and substantially below the PFAS flux of the Yangtze River to the sea (16.9 t/year; [101]). The monthly PFAS loads for the eight urban rivers were 19.34 and 5.53 kg, those for agricultural rivers were 2.95 and 1.57 kg, and those for restoration-type rivers were 2.46 and 1.69 kg, respectively. The PFAS inflow loads were higher in the wet season than in the dry season. Enhanced rainfall and runoff during the wet period not only transported greater quantities of terrestrial pollutants into the lake but also promoted contaminant release from the sediment–water interface through hydrodynamic disturbance [102]. In the wet season, urban tributaries such as the Luolong, Hai, Baoxiang, and Maliao Rivers exhibited higher PFAS loads than agricultural or restoration-type rivers, consistent with the spatial distribution of PFAS statistical hotspots. The elevated PFAS loads in urban rivers can be attributed to greater flow discharge and intensive anthropogenic activities. In contrast, during the dry season, the PFAS loads from restoration and agricultural rivers slightly exceeded those of urban rivers. Although urban rivers had higher contamination levels, their reduced flow limited total inflow loads, whereas agricultural and restoration rivers maintained relatively stable baseflow, resulting in slightly higher overall contributions. Source apportionment results confirmed that inflowing tributaries represent a major source of PFAS pollution in the lake. The quantitative estimation of PFAS inflow loads further substantiates this finding and identifies key tributaries requiring management attention during both wet and dry seasons.

Among the target PFASs, long-chain compounds accounted for 78.86% and 46.71% of the total mass load during the wet and dry seasons, respectively, while short-chain PFASs contributed 21.14% and 53.29% (Table S14). The calculated log *K_d_* indicates that long-chain PFASs exhibit stronger affinity for sediments. During the wet season, increased runoff and flow velocity promote sediment resuspension, thereby releasing adsorbed long-chain PFASs into the water column and elevating their inflow loads. In contrast, short-chain PFASs possess higher solubility and mobility, enabling continuous input to the lake via baseflow under low-flow conditions during the dry season, which consequently increases their relative contribution.

As a semi-enclosed lake, Dianchi is particularly susceptible to pollutant accumulation when external inputs exceed its self-purification capacity, potentially leading to water quality deterioration. Continuous monitoring of PFAS pollution within the basin is therefore essential to mitigate ecological risks and prevent bioaccumulation through the food chain that may pose threats to human health. This study estimated PFAS mass loads only for major inflowing rivers traversing large and medium-sized reservoirs within the Dianchi Basin, excluding smaller tributaries and seasonal channels. Given that such rivers may substantially contribute to pollutant fluxes during the wet season, uncertainties remain in the present load estimations. Moreover, interannual variations in flow, sampling period discrepancies, and analytical uncertainties may further influence the precision of PFAS load assessment. To enhance the representativeness and reliability of flux estimation, future studies should establish a more comprehensive watershed-scale monitoring framework integrating new pollutant surveillance with flow measurements.

### 3.4. PFASs’ Ecological Effects

**Figure 7:**
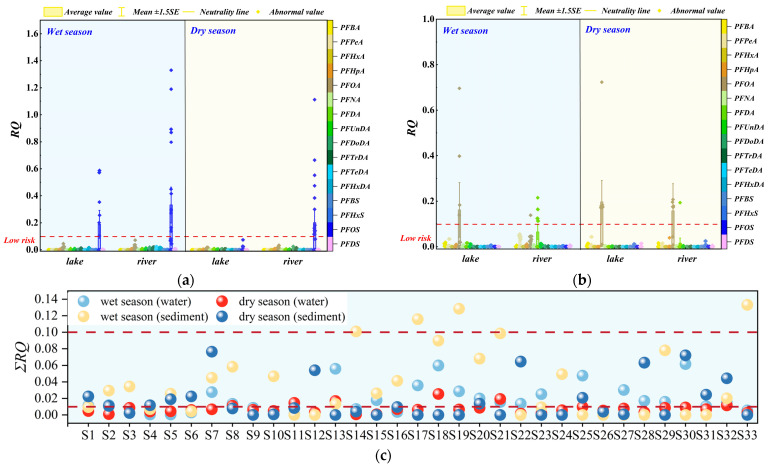
The risk quotient (*RQ*) values of PFASs in waters (**a**) and sediments (**b**) of the Dianchi Basin. Total risk quotient (∑*RQ*) during the wet and dry seasons (**c**).

This study calculated the *RQ* to evaluate the ecosystem effects of PFASs detected in the waters and sediments of the Dianchi Basin (see Figure 7). Overall, the results indicated that the *RQ* values in the waters were low (0.01), indicating that, under the current concentration range, long-term adverse effects on most aquatic organisms are unlikely, although continued monitoring is still recommended due to the persistence and bioaccumulative properties of PFASs. In sediments, the RQ values ranged from 0.01 to 1, at a low to moderate level of risk, suggesting potential for sublethal or chronic effects on sensitive species under long-term exposure and implying that source-control and precautionary management measures should be considered, especially in areas where multiple PFASs co-occur. Specifically, approximately 20% of river sediment points during the wet season exhibited moderate ecological risks of PFASs (0.1 < *RQ* < 1). Compared with other PFASs, PFPeA, PFDA, and PFOS in sediments pose more significant potential impacts on the ecosystem. This can be explained by their water–sediment partitioning characteristics. Long-chain compounds such as PFDA and PFOS exhibit higher partition coefficients (*K_d_* and *Koc*), indicating a stronger tendency to accumulate in sediments rather than remain in the water column. From the perspective of individual compounds, PFASs in both waters and sediments during the wet and dry seasons were generally associated with low risk levels. However, compared with other PFASs, PFPeA, PFDA, and PFOS in sediments exerted greater effects on the ecosystem. Previous studies have demonstrated that PFOS is the predominant contaminant in sediments in terms of both concentration and risk level [68,103], which is consistent with the findings of the present study. In general, the *RQ* values of PFASs in the basin ranged from 0.01 to 1, suggesting an overall low level of risk in the region. Nevertheless, source apportionment analysis indicated the presence of multiple PFAS-related pollution sources within the basin, including the textile, leather, metal plating, and plastic-processing industries. Although the overall ecological effects of PFASs are relatively low at present, the mass loads of PFASs transported from tributaries into the lake should not be overlooked. Therefore, continuous monitoring of potential ecological risks in the Dianchi Basin, particularly in the lacustrine ecosystem, is strongly recommended.

It should be noted that this study estimated the PFAS mass loads only for the major inflow rivers within the Dianchi Basin that pass through medium- and large-sized reservoirs, while several smaller tributaries and seasonal streams were not included in the sampling campaign. Because these rivers may contribute non-negligibly to the total pollutant flux during the wet season, the overall load estimation in this study likely involves a certain degree of underestimation. In addition, interannual variations in river discharge, temporal differences in sampling, and analytical uncertainties may further affect the accuracy of PFAS load estimation. Therefore, future studies should establish a more comprehensive river network monitoring system at the watershed scale to enhance the representativeness and reliability of PFAS flux assessments.

## 4. Conclusions

This study systematically characterized the contamination levels, source profiles, and ecological risks of PFASs in the surface waters and sediments of Dianchi Lake and its tributaries. The results showed that the PFAS concentrations in waters were moderate compared with those in other lakes in China, while the concentrations in sediments were comparable to those reported in recent studies of Dianchi Lake. Due to the restriction of long-chain PFASs, short-chain homologues accounted for a higher proportion in waters, whereas long-chain PFASs remained dominant in sediments. Distribution coefficient analysis indicated that long-chain PFASs and sulfonate-substituted PFASs were preferentially enriched in sediments, consistent with their stronger hydrophobicity and particle affinity. The primary sources of PFASs in river systems include industrial pollution from fluorinated polymer-processing agents, domestic pollution from food packaging and coating materials, mist suppressants used in metal electroplating, and atmospheric deposition of industrial emissions. In contrast, the main sources in the lake are related to mixed sources of consumer goods, atmospheric deposition, and riverine input, with the latter serving as the predominant source. Seasonal analysis revealed that the total PFAS mass loads from 12 major inflowing rivers were 24.75 kg in the wet season and 8.79 kg in the dry season, demonstrating persistent external inputs under varying hydrological conditions. Ecological risk assessment showed that PFASs in waters posed a low risk (0.01 < *RQ* < 0.1), while sediment exhibited low to moderate risk (0.1 < *RQ* < 1), with long-chain PFASs, particularly those with sulfonate groups, identified as the primary risk drivers. Overall, PFASs in the lake and its tributaries were present at moderate levels, comparable to previous findings in sediments. Although current ecological risks were generally low to moderate, the persistence of pollution sources and substantial tributary inputs highlights the need for continuous monitoring and targeted management. These findings provide a scientific basis for PFAS pollution control in the basin.

## Data Availability

The original contributions presented in this study are included in the article/Supplementary Materials. Further inquiries can be directed to the corresponding author.

## Supplementary Materials

The following supporting information can be downloaded at: https://www.mdpi.com/article/10.3390/toxics14030196/s1, Figure S1: The SWAT model analysis results for the wet season (A) and dry season (B). Flow Out denotes the average discharge of the outflow reach at each time step (m^3^/s); Figure S2: Correlation between PFASs in sediment and water. The significance level of is as follows: * *p* < 0.05, ** *p* < 0.01, *** *p* < 0.001; Figure S3: Cluster analysis of PFASs in water during the wet season (A) and dry season (B); Figure S4: Correlation between measured value and predicted value of PFASs source contribution rate in river water body; Figure S5: Industrial enterprises related to fluorine chemical production in Dianchi Basin; Figure S6: Spatial distribution of four river factors and superposition of geographical information; Figure S7: Correlation between measured value and predicted value of PFASs source contribution rate in lake water body; Figure S8: Spatial distribution of three lake factors and superposition of geographical information; Figure S9: The structural equation model used to describe the direct and indirect effects of administrative divisions, GDP per capita, population density, and the proportion of tertiary industry on ∑PFASs. (the width of the arrow indicates the strength of the standardized path coefficient (λ). A solid line represents a positive path coefficient, and a dashed line represents a negative path coefficient, The R^2^ value represents the proportion of variance explained by each endogenous variable CMIN/DF = the ratio of χ^2^ and degrees of freedom, GFI, goodness of fit index, RMSEA, the square root of the approximate error. The significance level of each path is as follows: * *p* < 0.05, ** *p* < 0.01, *** *p* < 0.001); Table S1: Information on sample sites for water bodies and sediments during the wet and dry seasons; Table S2: The group type, analyte formula, CAS number, acronym, and optimum LC-MS/MS parameters for multiple monitoring (MRM) acquisition conditions of 17 target per- and polyfluoroalkyl substances (PFASs) and 9 isotope-labeled substitute standards; Table S3: Gradient elution procedure for LC-MS/MS; Table S4: Coefficient of association (R2), recovery rates, recoveries of internal standards, limit of detections (LOD), limit of quantifications (LOQ) and blanks of the target PFASs in water; Table S5: Coefficient of association (R^2^), recovery rates, recoveries of internal standards, limit of detections (LOD), limit of quantifications (LOQ) and blanks of the target PFASs in sediment; Table S6: Measured values of water environmental factors during the wet season and dry season; Table S7: Lowest predicted no-effect concentrations (PNECs, μg/L) for selected PFASs in water and PNECs (μg/kg dry weight) in sediment; Table S8: Concentration value (ng/L) of 16 per- and polyfluoroalkyl substances (PFASs) in water of lakes and rivers in Dianchi Basin; Table S9: Concentration value (ng/g) of 16 per- and polyfluoroalkyl substances (PFASs) in sediment of lakes and rivers in Dianchi Basin; Table S10: Average log Kd and log Koc (L/kg) at sediment-water interface from Dianchi Basin in wet season; Table S11: Average log Kd and log Koc (L/kg) at sediment-water interface from Dianchi Basin in dry season; Table S12: Socio-economic development indicators of PFAS in different districts of Dianchi Basin; Table S13: The mass loads of 12 rivers flowing during the wet season and dry season; Table S14: The mass loads (kg/year) of 16 PFASs in wet season and dry season.

## Abbreviations

The following abbreviations are used in this manuscript:

PFASs	Per- and polyfluoroalkyl substances
PFOA	perfluorooctanoic acid
PFOS	perfluorooctanesulfonic acid
PMF	Positive Matrix Factorization
PCA	Principal Component Analysis
RDA	Redundancy analysis
PFBA	perfluorobutanoic acid
PFCA	perfluoroalkyl carboxylic acid groups
PFPeA	perfluoropentanoic acid
PFHxA	perfluorohexanoic acid
PFHpA	perfluoroheptanoic acid
PFNA	perfluorononanoic acid
PFDA	perfluorodecanoic acid
PFUnDA	perfluoroundecanoic acid
PFDoDA	perfluorododecanoic acid
PFTrDA	perfluorotridecanoic acid
PFTeDA	perfluorotetradecanoic acid
PFHxDA	perfluorohexadecanoic acid
PFODA	perfluorooctadecanoic acid
PFSA	perfluoroalkyl sulfonic acid groups
PFBS	perfluorobutane sulfonate
PFHxS	perfluorohexane sulfonate
PFDS	perfluorodecane sulfonate
MPFHxS	Methyl perfluorohexane sulfonate
MPFOS	Methyl perfluorooctane sulfonate
HPLC	High-performance liquid chromatography
SPE	solid-phase extraction
LOD	limit of detection
LOQ	limit of quantification
*C*	Concentration
SS	suspended solids
TN	total nitrogen
TP	total phosphorus
TOC	total organic carbon
Chla	chlorophyll a
COD	chemical oxygen demand
MDL	method detection limit
SWAT	Soil and Water Assessment Tool
*RQ*	risk quotient
∑*RQ*	total risk quotient
*MEC*	measured exposure concentration
*PNEC*	predicted no-effect concentration
CVs	coefficient of variation
EPA	U.S. Environmental Protection Agency
dw	dry weight
*K_d_*	partition coefficients
*Koc*	organic carbon-normalized partition coefficients
SEM	structural equation mode
