# Supplementary material for: Occurrence, Pollution Characteristics, Mass Load and Ecological Risk Assessment of Per- and Polyfluoroalkyl Substances in the Dianchi Basin, China
Source: Toxics. 2026 Feb 26;14(3):196. doi: 10.3390/toxics14030196 (PMC13030343; doi:10.3390/toxics14030196)
Supplement: Supplementary file 1 [file toxics-14-00196-s001.zip › toxics-4124533-supplementary.pdf]

Supplementary Materials

Occurrence, Pollution Characteristics, Mass Load and Ecological Risk Assessment of Per- and Polyfluoroalkyl Substances in the Dianchi Basin

Hongyi Liang [1], Tingting Ding [2], Yahui Zhang [3,*], Feng Miao [4], Zejun Wang [5], Shilin Du [6] and Jiale Cao [7]

[1] *Hebei Key Laboratory of Heavy Metal Deep-Remediation in Water and Resource Reuse, School of Environmental and Chemical Engineering, Yanshan University, Qinhuangdao 064004, China; 19932702423@163.com*

[2] *Environmental Analysis and Testing Laboratory, Chinese Research Academy of Environmental Sciences, Beijing 100012, PR China; dingtingting@craes.org.cn*

[3] *Environmental Analysis and Testing Laboratory, Chinese Research Academy of Environmental Sciences, Beijing 100012, PR China; zhangyahui@craes.org.cn*

[4] *Bayannur Substation of the Inner Mongolia Autonomous Region Environmental Monitoring Station, Inner Mongolia 015000, PR China; hongluohan@qq.com*

[5] *School of Environmental Science and Engineering, Suzhou University of Science and Technology, Suzhou, 215009, PR China; wangzj9135@163.com*

[6] *Environmental Analysis and Testing Laboratory, Chinese Research Academy of Environmental Sciences, Beijing 100012, PR China; du.shilin@craes.org.cn*

[7] *Beijing Zhonghe Intelligent Testing Technology Service Co., LTD, Beijing 102200, PR China; 17856929772 @163.com*

* Corresponding author at: State Key Laboratory of Environmental Criteria and Risk Assessment, Chinese Research Academy of Environmental Sciences, No. 8 Yard Dayangfang, Beijing 100012, PR China.

E-mail address: zhangyahui@craes.org.cn.

The supplementary materials contain 14 tables and 9 figures.

Table S1. Information on sample sites for water bodies and sediments during the wet and dry seasons. 1
Table S2. The group type, analyte formula, CAS number, acronym, and optimum LC-MS/MS parameters for multiple monitoring (MRM) acquisition conditions of 17 target per- and polyfluoroalkyl substances (PFASs) and 9 isotope-labeled substitute standards. 2
Table S3. Gradient elution procedure for LC-MS/MS. 5
Table S4. Coefficient of association (R^2), recovery rates, recoveries of internal standards, limit of detections (LOD), limit of quantifications (LOQ) and blanks of the target PFASs in water. 6
Table S5. Coefficient of association (R^2), recovery rates, recoveries of internal standards, limit of detections (LOD), limit of quantifications (LOQ) and blanks of the target PFASs in sediment. 8
Table S6. Measured values of water environmental factors during the wet season and dry season. 10
Table S7. Lowest predicted no-effect concentrations (*PNECs*, ug/L) for selected PFASs in water and *PNECs* (ug/kg dry weight) in sediment. 12
Table S8. Concentration value (ng/L) of 16 per- and polyfluoroalkyl substances (PFASs) in water of lakes and rivers in Dianchi Basin. 13
Table S9. Concentration value (ng/g) of 16 per- and polyfluoroalkyl substances (PFASs) in sediment of lakes and rivers in Dianchi Basin. 14
Table S10. Average log K_d and log K_{oc} (L/kg) at sediment-water interface from Dianchi Basin in wet season. 15
Table S11. Average log K_d and log K_{oc} (L/kg) at sediment-water interface from Dianchi Basin in dry season. 16
Table S12. Socio-economic development indicators of PFAS in different districts of Dianchi Basin. 17
Table S13. The mass loads of 12 rivers flowing during the wet season and dry season. 18
Table S14. The mass loads (kg/year) of 16 PFASs in wet season and dry season. 19
Fig. S1. The SWAT model analysis results for the wet season (A) and dry season (B). 21
Fig. S2. Correlation between PFASs in sediment and water. 22
Fig. S3. Cluster analysis of PFASs in water during the wet season (A) and dry season (B). 23
Fig. S4. Correlation between measured value and predicted value of PFASs source contribution rate in river water body. 24
Fig. S5. Industrial enterprises related to fluorine chemical production in Dianchi Basin. 25
Fig. S6. Spatial distribution of four river factors and superposition of geographical information. 26
Fig. S7. Correlation between measured value and predicted value of PFASs source contribution rate in lake water body. 27
Fig. S8. Spatial distribution of three lake factors and superposition of geographical information. 28
Fig. S9. The structural equation model used to describe the direct and indirect effects of administrative divisions, GDP per capita, population density, and the proportion of tertiary industry on ∑PFASs. 29

Table S1. Information on sample sites for water bodies and sediments during the wet and dry seasons.

Sample sites	Longitude	Latitude	Location	Water sample		Sediment sample	
				Wet season	Dry season	Wet season	Dry season
S1	102.69030	24.91360	Grey Bay	√	√	√	√
S2	102.74920	24.89000	Luojiaying	√	√	√	√
S3	102.70874	24.87395	Supplementary point	√	√	√	√
S4	102.67560	24.83720	Guanyin Shanxi	√	√	√	√
S5	102.70940	24.83720	Guanyin Shanzhong	√	√	√	√
S6	102.76110	24.83720	Guanyin Shandong	√	√	√	√
S7	102.84100	24.95896	Supplementary point	√	√	√	√
S8	102.67000	24.81000	Baiyukou	√	√	√	√
S9	102.66510	24.75648	Supplementary point	√	√		
S10	102.63000	24.77330	Haikou west	√	√	√	√
S11	102.63640	24.70640	South of Dianchi lake	√	√		√
S12	102.69667	24.95956	Panlongjiang	√	√		√
S13	102.73761	24.94476	Baoxiang river	√	√	√	
S14	102.77520	24.92300	Maliao river	√	√	√	√
S15	102.78690	24.88370	Luolong river	√	√	√	
S16	102.78380	24.82500	Laoyu river	√	√	√	√
S17	102.61460	24.66280	Gucheng river	√	√	√	
S18	102.58537	24.78814	Haikou river	√	√	√	
S19	102.67660	25.03179	Xinyunlianghe river	√	√	√	
S20	102.72086	25.08313	Panlong river	√	√	√	√
S21	102.76490	25.13827	Panlong river	√	√	√	
S22	102.84016	25.19683	Panlong river	√	√		√
S23	102.81689	25.36307	Panlong river	√	√	√	
S24	102.85689	25.41883	Panlong river	√	√	√	
S25	102.88525	25.30197	Dianwei river	√	√		√
S26	102.86266	25.22424	Dianwei river	√	√		√
S27	102.75627	24.99646	Haihe river	√	√		√
S28	102.79593	24.98849	Baoxianghe river	√	√		√
S29	102.72588	24.80216	Maliao river	√	√	√	
S30	102.88439	24.87556	Laoyu river	√	√		√
S31	102.73247	24.76386	Liangwang river	√	√		√
S32	102.71191	24.71499	Dahe river	√	√	√	√
S33	102.69856	24.64711	Dongdahe river	√	√	√	√

Table S2. The group type, analyte formula, CAS number, acronym, and optimum LC-MS/MS parameters for multiple monitoring (MRM) acquisition conditions of 17 target per- and polyfluoroalkyl substances (PFASs) and 9 isotope-labeled substitute standards.

Group type	Perfluoroalkyl Chain Length	Compound	CAS number	Ion Transition	Corresponding Internal Standard	Q1 Pre Bias (V)	CE (V)	Q3 Pre Bias (V)
PFCA	4	Perfluorobutyric acid (PFBA)	375-22-4	213>169	MPFBA	17	12	17
	5	Perfluorovaleric acid (PFPeA)	2706-90-3	263>219	MPFBA	20	7	24
	6	Perfluorohexanoic acid (PFHxA)	307-24-4	313>269	MPFHxA	24	10	32
	7	Perfluoroheptanoic acid (PFHpA)	375-85-9	363>319	MPFHxA	16	10	26
	8	Perfluorooctanoic acid (PFOA)	335-67- 1	413>369	MPFOA	16	11	22
	9	Perfluoroanonic acid (PFNA)	375-95- 1	463>419	MPFNA	18	11	30
	10	Perfluoroodecanoic acid (PFDA)	335-76-2	513>469	MPFDA	20	19	24
	11	Perfluorooundecanoic acid (PFUDA)	2058-94-8	563>519	MPFUdA	22	13	38
	12	Tricosafluorododecanoic acid (PFDoDA）	307-55- 1	613>569	MPFDoA	24	27	30
	13	Perfluoridecanoic acid (PFTrDA）	72629-94-8	663>619	MPFDoA	26	13	32
	14	Heptacosafluorotetradecanoic acid (PFTeDA）	376-06-7	713>669	MPFDoA	20	13	35

	16	Perfluorohexadecanoic acid (PFHxDA）	67905- 19-5	813.2>169	MPFDoA	20	14	40
	18	Perfluorooctadecanoic acid (PFODA)	16517- 11-6	913>869	MPFDoA	22	16	32
PFSA	4	Perfluorobenzene sulfonic acid (PFBS)	375-73-5	299>80	MPFHxS	22	33	30
	6	Perfluorohexane sulfonic acid (PFHxS)	355-46-4	399>98	MPFHxS	30	46	30
	8	Perfluorooctane sulfonic acid (PFOS)	1763-23- 1	499>79	MPFOS	20	48	28
	10	Perfluoroodecanesulfonic acid (PFDS)	335-77-3	599>80	MPFOS	20	55	30
		Perfluoro-n-[$^{13}C_4$] butanoic acid (MPFBA)	1017281-29-6	217>172	-	26	10	18
		Perfluoro-n-[1,2-$^{13}C_2$] hexanoic acid (MPFHxA)	960315-47-3	315>270	-	24	9	30
		Perfluoro-n-[1,2,3,4-$^{13}C_4$] octanoic acid (MPFOA)	960315-48-4	417>372	-	30	11	26
		Perfluoro-n-[1,2,3,4,5-$^{13}C_5$] nonanoic acid (MPFNA)	960315-49-5	468>423	-	18	11	30
		Perfluoro-n-[1,2-$^{13}C_2$] decanoic acid (MPFDA)	960315-50-8	515>470	-	20	12	23
		Perfluoro-n-[1,2-$^{13}C_2$] undecanoic acid (MPFUnDA)	960315-51-9	565>520	-	20	13	38
		Perfluoro-n-[1,2-$^{13}C_2$] dodecanoic acid (MPFDoDA)	960315-52-0	615>570	-	24	13	28

	Sodium perfluoro-1-[$^{18}O_2$] hexanesulfonic acid (MPFHxS)	1585941-14-5	403>103	-	15	35	19
	Sodium perfluoro-1-[1,2,3,4-$^{13}C_4$] octanesulfonic acid (MPFOS)	960315-53-1	503>80	-	36	50	30

Table S3. Gradient elution procedure for LC-MS/MS.

Time (min)	Mobile phase*		Flow
	A (%)	B (%)	(ml/min)
0.00	80	20	0.3
14.00	10	90	0.3
16.00	10	90	0.3
16.01	80	20	0.3
20.00	80	20	0.3

*: Solvent A was 2 mM ammonium acetate aqueous solution, solvent B was acetonitrile.

Table S4. Coefficient of association (R^2), recovery rates, recoveries of internal standards, limit of detections (LOD), limit of quantifications (LOQ) and blanks of the target PFASs in water.

Compound	R^2	Recovery rate (%)	Recoveries of internal standards % ±SD*	LOD (ng/L)	LOQ (ng/L)	Procedural blank (ng/L)	Travel blank (ng/L)
PFBA	0.992	90.21~109.82	-	0.03	0.09	No Peak	No Peak
PFPeA	0.997	103.72~120.56	-	0.09	0.29	No Peak	No Peak
MPFBA	-	-	87.16±11	-	-	-	-
PFHxA	0.999	89.31~112.54	-	0.05	0.17	<LOQ	No Peak
PFHpA	0.991	93.26~117.54	-	0.12	0.38	No Peak	No Peak
MPFHxA	-	-	92.03±9	-	-	-	-
PFOA	0.998	87.52~109.78	-	0.06	0.19	0.73±20	<LOQ
MPFOA	-	-	91.54±6	-	-	-	-
PFNA	0.995	90.80~106.55	-	0.05	0.16	No Peak	0.21±31
MPFNA	-	-	79.81±12	-	-	-	-
PFDA	0.998	79.03~101.28	-	0.01	0.04	No Peak	<LOQ
MPFDA	-	-	87.00±5	-	-	-	-
PFUDA	0.999	80.22~96.11	-	0.03	0.09	No Peak	No Peak
MPFUDA	-	-	83.09±14	-	-	-	-
PFDoDA	0.994	79.01~102.38	-	0.14	0.43	No Peak	No Peak
PFTrDA	0.996	85.67~113.86	-	0.17	0.55	No Peak	No Peak
PFTeDA	0.999	90.59~108.57	-	0.19	0.61	No Peak	No Peak
PFHxDA	0.999	92.10~117.93	-	0.11	0.34	No Peak	No Peak
PFODA	0.998	104.39~119.03	-	0.13	0.40	No Peak	No Peak
MPFDoA	-	-	93.75±9	-	-	-	-
PFBS	0.999	87.30~107.86	-	0.11	0.34	No Peak	No Peak
PFHxS	0.994	98.87~116.73	-	0.13	0.41	No Peak	No Peak

MPFHxS	-	-	74.94±11	-	-	-	-
PFOS	9.999	92.31±8	-	0.10	0.31	No Peak	No Peak
PFDS	0.999	90.22~113.58	-	0.11	0.36	No Peak	No Peak
MPFOS	-	-	82.59±4	-	-	-	-

*: Standard deviation.

Table S5. Coefficient of association (R^2), recovery rates, recoveries of internal standards, limit of detections (LOD), limit of quantifications (LOQ) and blanks of the target PFASs in sediment.

Compound	R^2	Recovery rate (%)	Recoveries of internal standards %±SD	LOD (ng/g)	LOQ (ng/g)	Procedural blank (ng/g)	Travel blank (ng/g)
PFBA	0.994	92.90~112.37	-	0.02	0.08	No Peak	No Peak
PFPeA	0.990	107.64~124.93	-	0.10	0.32	No Peak	No Peak
MPFBA	-	-	87.51±6	-	-	-	-
PFHxA	0.999	83.26~109.80	-	0.02	0.07	No Peak	No Peak
PFHpA	0.992	99.47~119.05	-	0.04	0.14	No Peak	No Peak
MPFHxA	-	-	91.30±8	-	-	-	-
PFOA	0.990	88.62~114.70	-	0.07	0.23	<LOQ	No Peak
MPFOA	-	-	79.69±7	-	-	-	-
PFNA	0.990	94.70~116.41	-	0.03	0.11	No Peak	No Peak
MPFNA	-	-	86.43±5	-	-	-	-
PFDA	0.999	81.26~118.33	-	0.03	0.09	No Peak	No Peak
MPFDA	-	-	77.71±14	-	-	-	-
PFUDA	0.997	93.22~97.87	-	0.05	0.15	No Peak	No Peak
MPFUDA	-	-	84.52±9	-	-	-	-
PFDoDA	0.990	78.41~92.30	-	0.08	0.26	No Peak	No Peak
PFTrDA	0.999	89.17~108.25	-	0.17	0.56	No Peak	No Peak
PFTeDA	0.994	92.59~119.57	-	0.02	0.07	No Peak	No Peak
PFHxDA	0.998	108.29~117.93	-	0.13	0.43	No Peak	No Peak
PFODA	0.996	109.34~119.03	-	0.08	0.28	No Peak	No Peak
MPFDoA	-	-	81.80±6	-	-	-	-
PFBS	0.992	83.20~112.36	-	0.05	0.15	No Peak	No Peak
PFHxS	0.993	90.15~109.23	-	0.07	0.23	No Peak	No Peak

MPFHxS	-	-	89.22±5	-	-	-	-
PFOS	9.995	84.41~91.05	-	0.10	0.33	No Peak	No Peak
PFDS	0.992	94.22~118.56	-	0.14	0.45	No Peak	No Peak
MPFOS	-	-	83.74±10	-	-	-	-

Table S6. Measured values of water environmental factors during the wet season and dry season.

Sample	SS*		Chla*		COD*		TN*		TP*		TOC*		*foc**	
	Dry season	Wet season	Dry season	Wet season	Dry season	Wet season	Dry season	Wet season	Dry season	Wet season	Dry season	Wet season	Dry season	Wet season
S1	0.002	0.042	21.990	1.990	28.370	28.370	1.350	0.350	0.045	0.045	4.840	4.840	5.085	2.930
S2	0.004	0.014	11.800	6.630	25.780	25.780	0.270	1.270	0.028	0.028	11.920	8.920	4.892	3.432
S3	0.305	0.045	30.260	2.210	33.560	33.560	1.470	1.470	0.042	0.042	5.020	5.020	4.984	3.608
S4	0.016	0.016	50.990	12.210	21.330	21.330	1.020	1.020	0.061	0.061	8.470	8.470	6.085	3.632
S5	0.006	0.006	1.460	4.050	20.590	20.590	1.930	1.930	0.036	0.096	4.020	4.020	7.718	2.878
S6	0.021	0.021	7.200	6.630	26.150	26.150	2.030	0.030	0.044	0.094	38.330	4.330	5.180	3.342
S7	0.023	0.023	24.010	2.210	19.850	19.850	2.160	0.160	0.048	0.048	1.880	5.880	5.993	3.477
S8	0.021	0.021	6.270	12.210	21.330	21.330	1.820	1.820	0.037	0.037	1.050	4.050	6.699	3.406
S9	0.022	0.022	5.680	5.210	16.520	16.520	1.940	1.940	0.045	0.045	17.850	7.850	-	-
S10	0.323	0.023	23.670	9.250	18.000	18.000	1.520	1.520	0.049	0.049	4.960	1.960	8.115	3.302
S11	0.017	0.017	5.270	10.367	15.410	5.410	1.410	1.410	0.034	0.094	47.770	4.770	-	3.455
S12	0.316	0.016	11.190	10.000	0.000	0.000	1.465	0.465	0.108	0.028	1.755	10.755	-	3.130
S13	0.017	0.017	2.050	7.364	7.630	7.630	6.177	1.177	0.039	0.039	9.467	8.467	2.766	-
S14	0.028	0.028	6.630	8.498	16.150	6.150	2.379	0.379	0.032	0.032	11.491	1.491	5.776	3.862
S15	0.028	0.028	20.210	6.157	0.000	0.000	1.388	1.388	0.025	0.025	8.464	1.464	4.322	-
S16	0.031	0.231	20.210	5.231	0.000	0.000	1.369	0.369	0.048	0.048	9.912	0.912	8.230	4.079
S17	0.009	0.019	2.210	4.024	0.000	0.000	0.927	1.927	0.101	0.031	4.289	0.289	7.563	-
S18	0.211	0.011	6.250	6.331	12.810	12.810	1.687	1.687	0.069	0.069	11.449	1.449	8.256	-
S19	0.000	0.000	1.367	5.041	2.810	12.810	1.410	1.410	0.049	0.049	1.966	0.966	7.710	-
S20	0.707	0.007	41.759	4.759	0.000	0.000	1.465	1.465	0.034	0.034	55.711	0.711	5.002	6.076
S21	0.016	0.016	1.759	8.759	0.000	0.000	0.177	1.177	0.108	0.080	1.033	1.033	3.220	-
S22	0.303	0.003	2.050	6.315	10.590	10.590	2.379	0.379	0.039	0.039	2.729	0.729	-	5.640

S23	0.603	0.803	11.630	7.112	2.440	12.440	0.388	0.388	0.032	0.032	9.656	12.656	5.151	-
S24	0.210	0.010	9.210	6.284	0.000	0.000	0.369	1.369	0.025	0.025	2.755	2.755	6.054	-
S25	0.019	0.019	2.210	7.016	0.220	10.220	2.160	0.160	0.048	0.048	13.467	2.467	-	3.620
S26	0.307	0.007	2.210	8.001	2.440	2.440	1.820	0.820	0.101	0.091	5.491	5.491	-	3.625
S27	0.007	0.007	48.250	8.250	6.520	16.520	1.940	1.940	0.069	0.069	2.464	2.464	-	5.282
S28	0.322	0.022	1.367	5.367	8.740	8.740	1.520	0.520	0.042	0.142	2.912	4.912	-	6.718
S29	0.315	0.415	22.719	40.719	15.780	15.780	1.410	1.410	0.061	0.061	4.289	6.289	3.661	-
S30	0.614	0.000	1.364	7.364	9.480	9.480	1.465	1.465	0.036	0.136	0.449	0.449	-	3.966
S31	0.001	0.201	2.498	8.498	3.930	13.930	0.369	1.369	0.044	0.044	11.766	5.766	-	6.914
S32	0.003	0.203	1.157	6.157	2.070	12.070	0.927	2.927	0.048	0.048	4.491	3.491	4.746	6.132
S33	0.004	0.304	1.157	6.157	0.000	0.000	1.687	1.687	0.037	0.137	2.033	2.033	5.085	2.930

*: Six key water environmental factors suspended solids (SS), total nitrogen (TN, mg/L), total phosphorus (TP, mg/L), total organic carbon (TOC, mg/L), chlorophyll (Chla, μg/L), chemical oxygen demand (COD, mg/L), and organic carbon fraction of sediments (f_{oc}, %).

Table S7. Lowest predicted no-effect concentrations (*PNECs*, ug/L) for selected PFASs in water and *PNECs* (ug/kg dry weight) in sediment.

Compound	$PNEC_{Water}$ ug/L	$PNEC_{Sediment}$ ug/kg dw
PFBA	27.8	166
PFPeA	3.91	32
PFHxA	140	7602
PFHpA	0.5	53.5
PFOA	98	2060
PFNA	1	142
PFDA	0.17	11
PFUnDA	0.13	103
PFDoDA	0.11	485
PFTrDA	0.1	940
PFTeDA	0.08	954
PFHxDA	0.08	583
PFODA	0.07	453
PFBS	372	2166
PFHxS	0.87	101
PFOS	25	67
PFDS	0.16	154

The Lowest *PNECs* value obtained from NORMAN Ecotoxicology Database.
https:/hwww.norman-network.com'nds/ecotoxlowestPnecsIndex.php

Table S8. Concentration value (ng/L) of 16 per- and polyfluoroalkyl substances (PFASs) in water of lakes and rivers in Dianchi Basin.

PFASs		Wet season								Dry season							
		Lake				River				Lake				River			
		Range	Mean	DF*	CV**	Range	Mean	DF	CV	Range	Mean	DF	CV	Range	Mean	DF	CV
PFCA	PFBA	ND~5.23	1.68	73	1.10	ND~5.69	1.26	82	1.07	ND~6.37	1.27	55	1.84	ND~7.49	1.29	50	1.73
	PFPeA	ND~1.17	0.27	55	1.37	ND~0.57	0.15	68	1.07	ND~0.36	0.13	55	1.18	ND~0.46	0.13	41	1.42
	PFHxA	ND~1.07	0.20	64	1.64	ND~1.38	0.19	50	1.67	ND~0.46	0.07	45	2.00	ND~0.51	0.07	32	1.98
	PFHpA	ND~2.03	0.32	27	2.15	ND~3.93	0.76	59	1.37	ND~1.79	0.38	36	1.63	ND~3.93	1.10	55	1.28
	PFOA	ND~8.07	1.66	94	1.54	ND~12.73	1.86	88	1.58	ND~3.12	1.01	64	1.04	ND~5.91	0.74	73	1.92
	PFNA	ND~0.82	0.14	18	2.23	ND~1.97	0.46	36	1.56	ND~1.21	0.21	45	1.76	ND~1.26	0.18	50	2.03
	PFDA	ND~1.53	0.19	36	2.41	ND~1.26	0.19	41	1.82	ND~1.36	0.31	55	1.54	ND~0.77	0.20	55	1.17
	PFUnDA	ND~1.10	0.12	18	2.86	ND~1.64	0.16	36	2.42	ND~0.55	0.14	27	1.73	ND~0.53	0.08	27	2.08
	PFDoDA	ND~0.25	0.05	36	1.75	ND~2.10	0.60	68	1.36	ND~0.72	0.11	45	2.02	ND~2.60	0.18	55	3.07
	PFTrDA	ND~1.17	0.17	27	2.09	ND~1.99	0.42	41	1.50	ND~0.65	0.18	36	1.48	ND~1.42	0.17	32	2.02
	PFTeDA	ND	-	-	-	ND~1.85	0.30	23	1.95	ND	-	-	-	ND	-	-	-
	PFHxDA	ND~1.13	0.13	27	2.56	ND~1.28	0.27	36	1.61	ND	-	-	-	ND~0.25	0.01	5	4.69
PFSA	PFBS	ND~5.25	1.64	82	1.19	ND~7.35	0.75	73	2.07	ND~6.00	1.48	82	1.22	ND~11.17	1.90	45	1.62
	PFHxS	ND~1.18	0.24	55	1.50	ND~1.34	0.21	50	1.75	ND~0.36	0.06	18	2.23	ND~0.38	0.07	36	1.55
	PFOS	ND~1.17	0.38	55	1.21	ND~2.66	0.66	68	1.25	ND~0.15	0.04	36	1.63	ND~2.22	0.40	59	1.41
	PFDS	ND~0.46	0.07	27	1.99	ND~1.20	0.17	32	1.89	ND~0.11	0.11	9	3.32	ND~0.31	0.01	5	4.69
ΣPFAS		ND~11.21	7.25	-	-	ND~20.79	8.42	-	-	ND~9.49	5.34	-	-	ND~15.67	6.43	-	-

*DF refers to detection rate (%).

**CV refers to coefficient of variation.

Table S9. Concentration value (ng/g) of 16 per- and polyfluoroalkyl substances (PFASs) in sediment of lakes and rivers in Dianchi Basin.

PFASs		Wet season								Dry season							
		Lake				River				Lake				River			
		Range	Mean	DF	CV	Range	Mean	DF	CV	Range	Mean	DF	CV	Range	Mean	DF	CV
PFCA	PFBA	0.03~3.19	1.12	100	0.99	ND~1.04	0.21	79	1.51	ND~2.49	0.68	50	1.30	ND~2.71	0.41	31	2.11
	PFPeA	ND~0.84	0.15	78	1.80	ND~1.36	0.70	79	0.77	ND~1.07	0.19	80	1.71	ND~0.39	0.06	23	2.21
	PFHxA	0.05~0.96	0.17	100	1.77	ND~1.09	0.23	93	1.25	ND~1.07	0.33	80	1.04	ND~0.84	0.17	31	1.91
	PFHpA	ND~0.50	0.07	22	2.40	ND~0.61	0.20	50	1.17	ND	-	-	-	ND~2.10	0.16	8	3.61
	PFOA	ND~4.20	0.98	67	1.48	ND~0.84	0.18	79	1.25	0.04~4.37	1.19	100	1.00	ND~6.42	0.95	62	1.82
	PFNA	ND~1.10	0.14	56	2.57	ND~1.14	0.20	43	1.67	ND~0.52	0.15	50	1.43	ND~0.60	0.20	54	1.16
	PFDA	ND~0.06	0.01	11	3.00	ND~0.76	0.23	43	1.25	ND	-	-	-	ND~0.68	0.06	15	3.42
	PFUnDA	ND~1.24	0.22	33	1.99	ND~1.40	0.45	50	1.29	ND~0.23	0.07	30	1.61	ND~0.44	0.04	15	2.88
	PFDoDA	ND~0.43	0.12	44	1.36	ND~1.76	0.17	43	2.68	ND~0.14	0.03	30	1.84	ND~0.13	0.03	23	1.90
	PFTrDA	ND~0.44	0.06	22	2.65	ND~3.32	0.62	64	1.45	ND~0.48	0.11	40	1.48	ND~1.67	0.18	31	2.61
	PFTeDA	ND~0.54	0.06	22	2.79	ND~1.30	0.42	50	1.20	ND~0.12	0.01	10	3.16	ND	-	-	-
	PFHxDA	ND~0.23	0.03	11	3.00	ND~0.53	0.13	43	1.45	ND	-	-	-	ND~0.04	0.04	8	3.61
PFSA	PFBS	ND~2.68	0.75	56	1.24	ND~2.07	0.60	79	1.04	ND~2.14	0.74	60	1.16	ND~3.41	0.78	38	1.49
	PFHxS	ND~0.67	0.10	22	2.21	ND~0.44	0.13	71	0.92	ND~1.19	0.21	30	1.93	ND~2.51	0.32	31	2.27
	PFOS	ND~2.26	0.70	56	1.41	ND~3.81	0.78	71	1.39	ND~0.85	0.14	30	2.14	ND~2.87	0.67	31	1.73
	PFDS	ND~0.12	0.01	11	3.00	ND~1.03	0.20	29	1.73	ND~2.20	0.22	10	3.16	ND~0.93	0.10	15	2.74
ΣPFAS		ND~10.21	4.67	-	-	ND~9.63	5.41	-	-	ND~11.47	3.98	-		ND~9.93	4.12	-	-

Table S10. Average log K_d and log K_{oc} (L/kg) at sediment-water interface from Dianchi Basin in wet season.

Type	Perfluoroalkyl Chain Length (n carbon atoms)	Lake		River	
		logK_d	logK_{oc}	logK_d	logK_{oc}
PFCA	4	1.03±0.19	2.02±0.19	1.53±0.19	2.02±0.19
	5	1.02±0.12	2.08±0.16	1.72±0.12	2.08±0.16
	6	1.08±0.23	2.17±0.18	1.67±0.23	2.17±0.18
	7	1.28±0.12	2.10±0.13	-	-
	8	1.96±0.25	2.54±0.23	2.55±0.16	3.00±0.16
	9	1.55±0.26	3.00±0.26	2.53±0.21	4.17±0.17
	10	-	-	-	-
	11	2.13±0.01	3.19±0.21	2.10±0.10	4.19±0.12
	12	-	-	-	-
	13	2.59±0.40	3.19±0.30	2.59±0.40	3.19±0.30
	16	2.90±0.12	3.21±0.33	-	-
	18	-	-	-	-
	Linear equation	Y= 0.1744x+0.2063 R^2= 0.9311	Y= 0.1285x+1.5469 R^2=0.7219	Y=0.0774x+1.2480 R^2=0.3296	Y=0.2513x+0.9735 R^2=0.4835
PFSA	4	1.03±0.24	3.73±0.47	1.73±0.37	4.73±0.13
	6	1.91±0.37	3.31±0.07	1.81±0.27	4.31±0.07
	8	1.40±0.30	3.24±0.53	2.40±0.53	4.24±0.13
	10	2.78±0.19	3.38±0.40	-	4.38±0.03
	Linear equation	Y0.2365x+0.1267 R^2=0.6497	Y=-0.0568x+3.8145 R^2=0.4531	Y=0.0811x+1.4384 R^2=0.4835	Y=-0.0569x+4.8145 R^2=0.4531

Table S11. Average log K_d and log K_{oc} (L/kg) at sediment-water interface from Dianchi Basin in dry season.

Type	Perfluoroalkyl Chain Length (n carbon atoms)	Lake		River	
		logK_d	logK_{oc}	logK_d	logK_{oc}
PFCA	4	3.53±0.29	1.03±0.09	2.53±0.32	1.02±0.09
	5	3.12±0.24	1.07±0.14	2.12±0.22	1.08±0.16
	6	3.17±0.23	1.14±0.13	2.17±0.28	1.17±0.38
	7	3.25±0.39	1.18±0.09	-	-
	8	4.14±0.25	1.19±0.05	2.54±0.52	1.54±0.52
	9	3.76±0.16	1.25±0.16	2.76±0.46	1.55±0.42
	10	-	-	-	-
	11	3.85±0.21	1.31±0.02	3.29±0.27	1.19±0.17
	12	-	-	-	-
	13	3.92±0.40	1.29±0.19	3.29±0.42	1.19±0.17
	16	3.80±0.23	1.31±0.12	-	-
	18	-	-	-	-
	Linear equation	Y= 0.0563x+3.1201 R^2= 0.3681	Y= 0.0180x+1.0421 R^2= 0.6280	Y=0.1040x+1.7523 R2=0.6732	Y= 0.0561x+0.8175 R^2= 0.5240
PFSA	4	3.73±0.37	1.51±0.17	3.73±0.27	1.73±0.41
	6	3.81±0.27	1.47±0.17	3.31±0.17	1.31±0.23
	8	3.40±0.53	1.69±0.13	3.24±0.33	2.24±0.13
	10	4.08±0.29	1.66±0.29	3.88±0.41	2.38±0.20
	Linear equation	Y=0.03118x+3.5380 R^2=0.0828	Y=0.0335x+1.3513 R^2=0.6327	Y=0.0180x+3.4145 R2=0.0225	Y=0.1431x+0.9145 R^2=0.5737

Table S12. Socio-economic development indicators of PFAS in different districts of Dianchi Basin.

Sampling district	GDP per capita (ten thousand yuan)	Population density (person/km^2)	Proportion of tertiary industry (%)
Guandu district	162.31	66.00	91157
Chenggong district	69.47	57.90	86926
Jinning district	34.88	59.30	57849
Xishan district	97.85	84.10	108678
Panlong district	102.27	77.10	106497

Table S13. The mass loads of 12 rivers flowing during the wet season and dry season.

River	Sample sites	Flow rate (m^3/s)		Mass loads (kg)	
		Wet season	Dry season	Wet season	Dry season
Panlong river	S12, S20-S24	38.96	17.92	1.97	0.38
Baoxiang river	S13, S28	53.12	22.43	2.56	0.48
Maliao river	S14, S29	2.01	0.93	2.10	0.86
Luolong river	S15	96.95	40.76	5.22	0.21
Laoyu river	S16, S30	50.18	21.13	1.69	0.84
Gucheng river	S17	3.11	1.36	1.85	0.33
Haikou river	S18	97.08	40.79	2.78	1.75
Xinyunlianghe river	S19	96.95	40.76	1.17	0.67
Daqing river	S25, S26	4.57	2.11	1.42	1.38
Hai river	S27	97.08	40.79	2.78	1.75
Nanchong river	S31	3.11	1.36	1.05	0.31
Dahe river	S32	3.11	1.36	2.09	1.01
Dongdahe river	S33	6.09	2.70	0.86	0.56

Table S14. The mass loads (kg/year) of 16 PFASs in wet season and dry season.

Period	Sample sites	PFBA	PFPeA	PFHxA	PFHpA	PFOA	PFNA	PFDA	PFUnDA	PFDoDA	PFTrDA	PFTe[illegible]
Wet season	S12	0.27	0.03	/	0.05	0.95	/	/	/	0.03	/	/
	S13	0.06	0.04	/	0.65	0.32	/	0.06	0.09	0.53	0.36	/
	S14	0.50	0.03	/	/	/	/	/	0.13	/	/	/
	S15	0.65	/	0.20	0.29	3.20	0.16	/	/	/	/	[illegible]
	S16	0.52	0.10	/	0.41	/	/	/	/	/	/	/
	S17	0.04	/	0.04	/	0.41	0.03	/	0.41	0.03	0.16	/
	S18	0.22	0.04	/	/	0.38	0.39	0.14	0.20	0.34	0.24	[illegible]
	S19	0.01	/	0.04	0.01	/	0.35	/	0.00	0.32	0.38	/
	S20	1.43	0.03	/	0.23	/	/	0.26	0.00	0.29	/	/
	S21	0.14	0.03	0.05	0.15	0.44	/	/	0.00	0.26	0.08	/
	S22	0.07	0.02	0.10	0.00	0.72	/	/	0.01	0.27	0.00	/
	S23	0.38	0.06	/	0.15	/	/	/	0.01	0.01	0.18	/
	S24	0.34	0.14	/	0.00	0.18	/	0.01	/	/	/	/
	S25	0.03	/	0.03	0.00	0.23	/	0.32	/	0.32	/	[illegible]
	S26	/	/	/	0.22	0.34	/	0.04	/	/	/	/
	S27	1.43	0.03	0.04	0.49	0.99	0.39	0.00	0.01	0.28	0.32	/
	S28	0.03	/	/	/	0.20	0.44	0.08	0.02	0.27	0.07	/
	S29	0.82	0.12	/	/	1.63	/	/	/	/	/	[illegible]
	S30	/	0.09	0.09	/	/	/	0.10	/	/	0.50	[illegible]
	S31	0.04	/	0.11	0.14	/	0.50	/	/	0.21	/	/
	S32	/	0.03	/	0.99	0.31	0.32	0.05	/	0.14	/	/
	S33	/	0.04	0.02	0.41	/	/	/	/	0.01	/	/
Dry season	S12	0.02	/	/	/	0.08	0.01	0.04	/	0.01	/	/
	S13	/	0.04	/	0.05	0.04	0.00	0.04	0.05	0.01	0.06	/
	S14	/	0.04	0.01	/	0.01	0.00	/	/	0.01	/	/
	S15	0.10	0.04	/	0.04	/	/	/	/	/	/	/
	S16	/	/	/	/	0.00	/	0.05	0.05	0.01	/	/
	S17	0.21	0.01	0.01	/	0.04	/	/	/	/	0.07	/
	S18	0.75	/	/	/	0.62	0.04	0.01	/	0.27	/	/
	S19	0.33	0.04	0.02	0.12	/	0.08	0.05	/	0.01	/	/
	S20	/	/	/	/	/	/	0.08	0.06	/	/	/
	S21	/	/	/	0.18	0.03	/	/	/	/	0.15	/
	S22	0.14	0.02	0.02	/	/	/	/	/	0.02	/	/
	S23	/	/	/	/	0.07	0.01	0.03	/	/	/	/
	S24	0.22	0.01	0.04	0.19	/	/	/	0.01	0.01	/	/
	S25	0.40	0.05	0.01	0.29	0.04	0.01	/	/	/	0.04	/
	S26	/	/	/	0.00	0.15	/	0.06	/	0.02	/	/
	S27	/	/	/	0.41	0.00	/	0.00	/	/	/	/
	S28	/	/	/	/	/	/	0.02	0.01	/	0.01	/
	S29	0.79	/	/	0.20	0.36	0.13	/	/	/	0.03	/

S30	0.03	0.04	0.05	0.38	0.09	0.02	0.03	/	/	/	/
S31	/	/	/	0.17	/	/	/	0.01	/	0.03	/
S32	0.01	/	/	0.42	0.08	0.00	0.04	/	0.02	/	/
S33	/	/	/	/	0.00	0.12	/	/	0.02	/	/

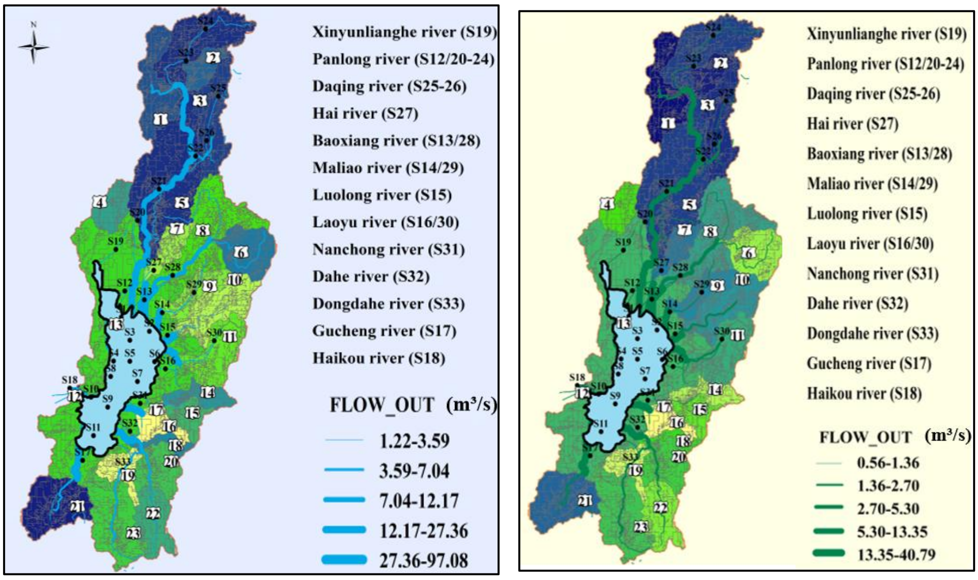

(A) (B)

Fig. S1. The SWAT model analysis results for the wet season (A) and dry season (B). Flow Out denotes the average discharge of the outflow reach at each time step (m^3/s).

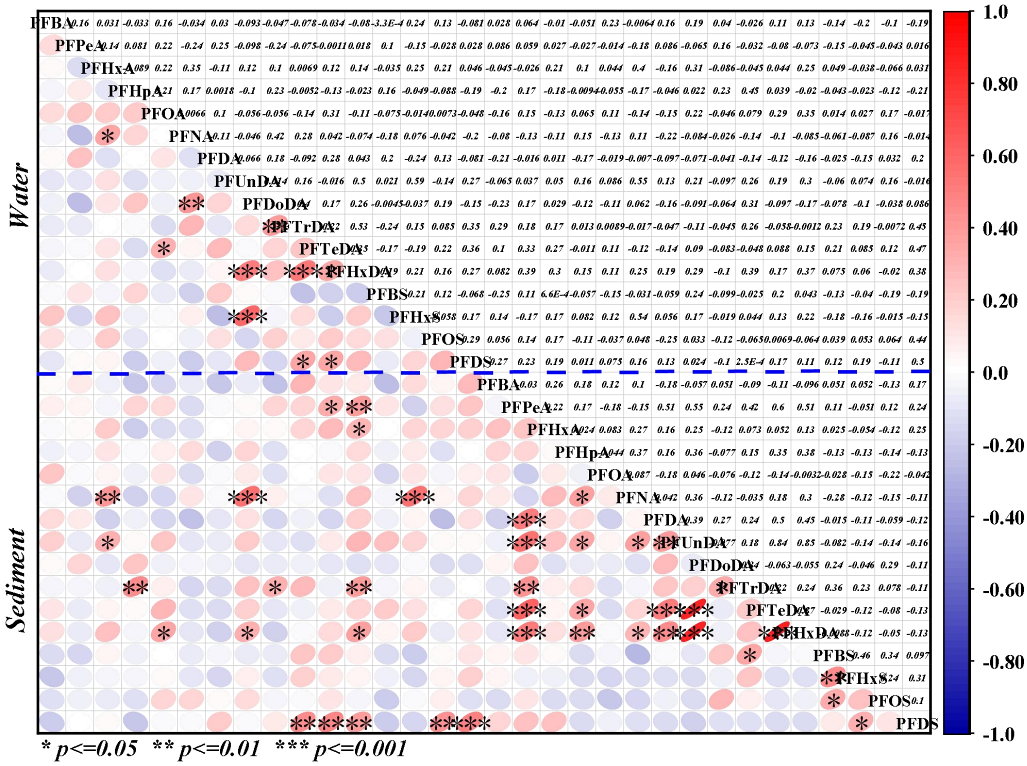

Fig. S2. Correlation between PFASs in sediment and water. The significance level of is as follows: *p<0.05, **p< 0.01, ***p <0.001.

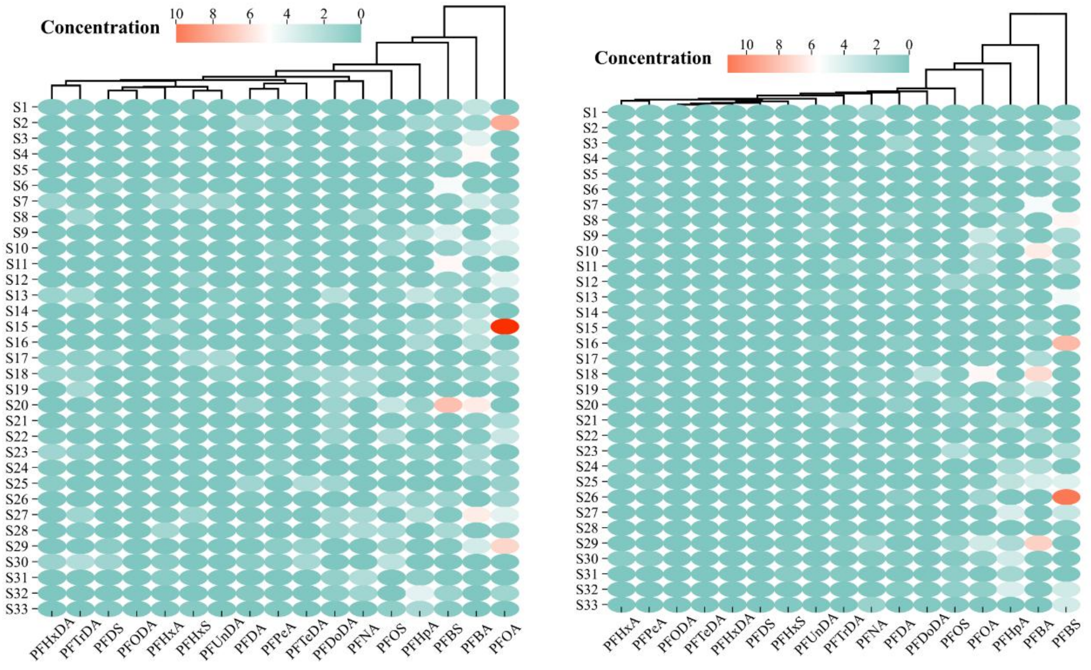

(A) (B)

Fig. S3. Cluster analysis of PFASs in water during the wet season (A) and dry season (B).

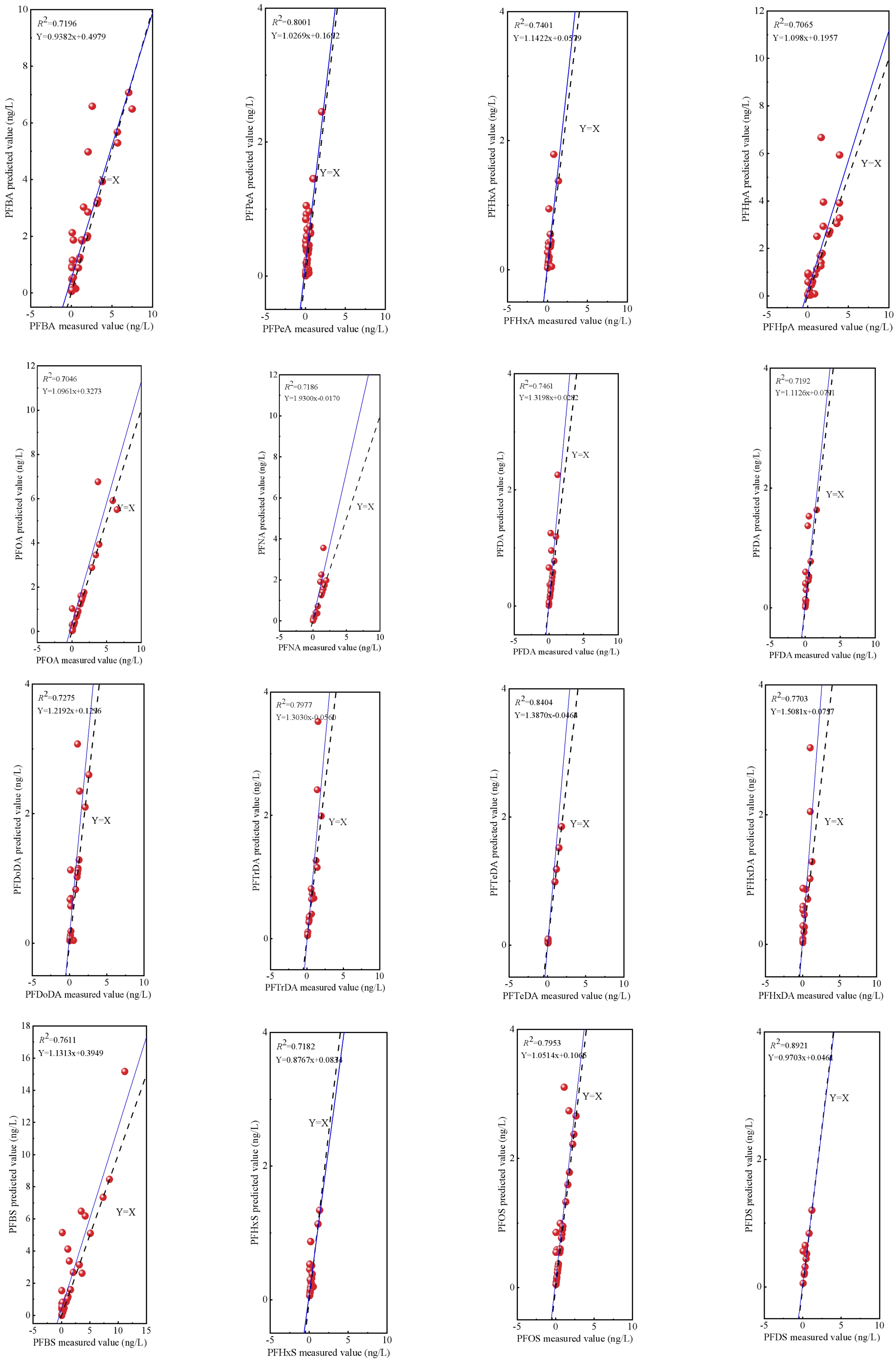

Fig. S4. Correlation between measured value and predicted value of PFASs source contribution rate in river water body.

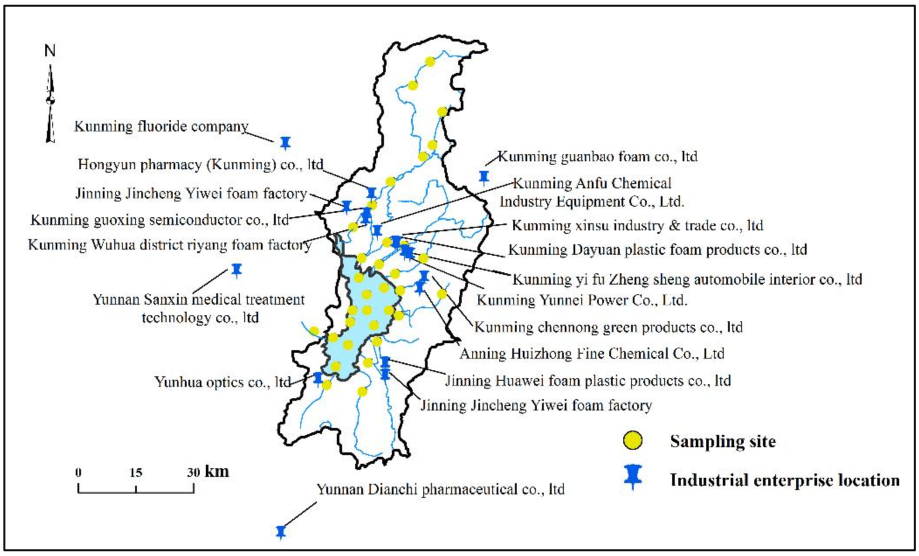

Fig. S5. Industrial enterprises related to fluorine chemical production in Dianchi Basin.

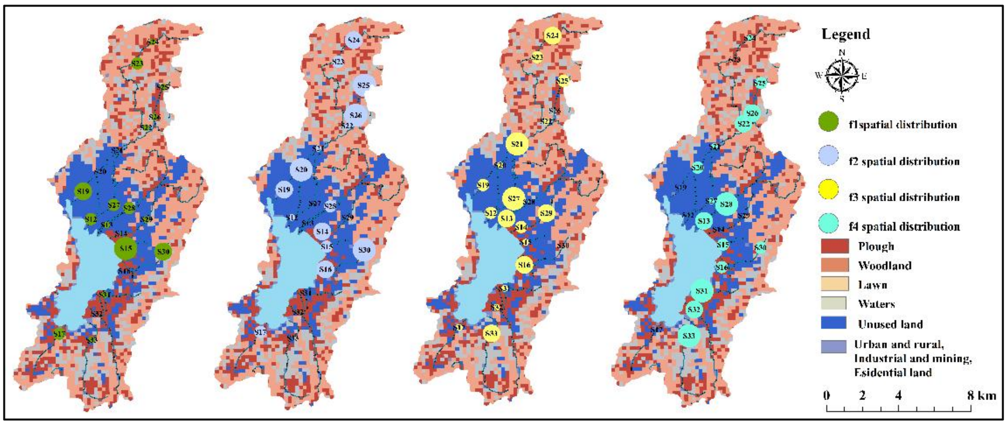

Fig. S6. Spatial distribution of four river factors and superposition of geographical information.

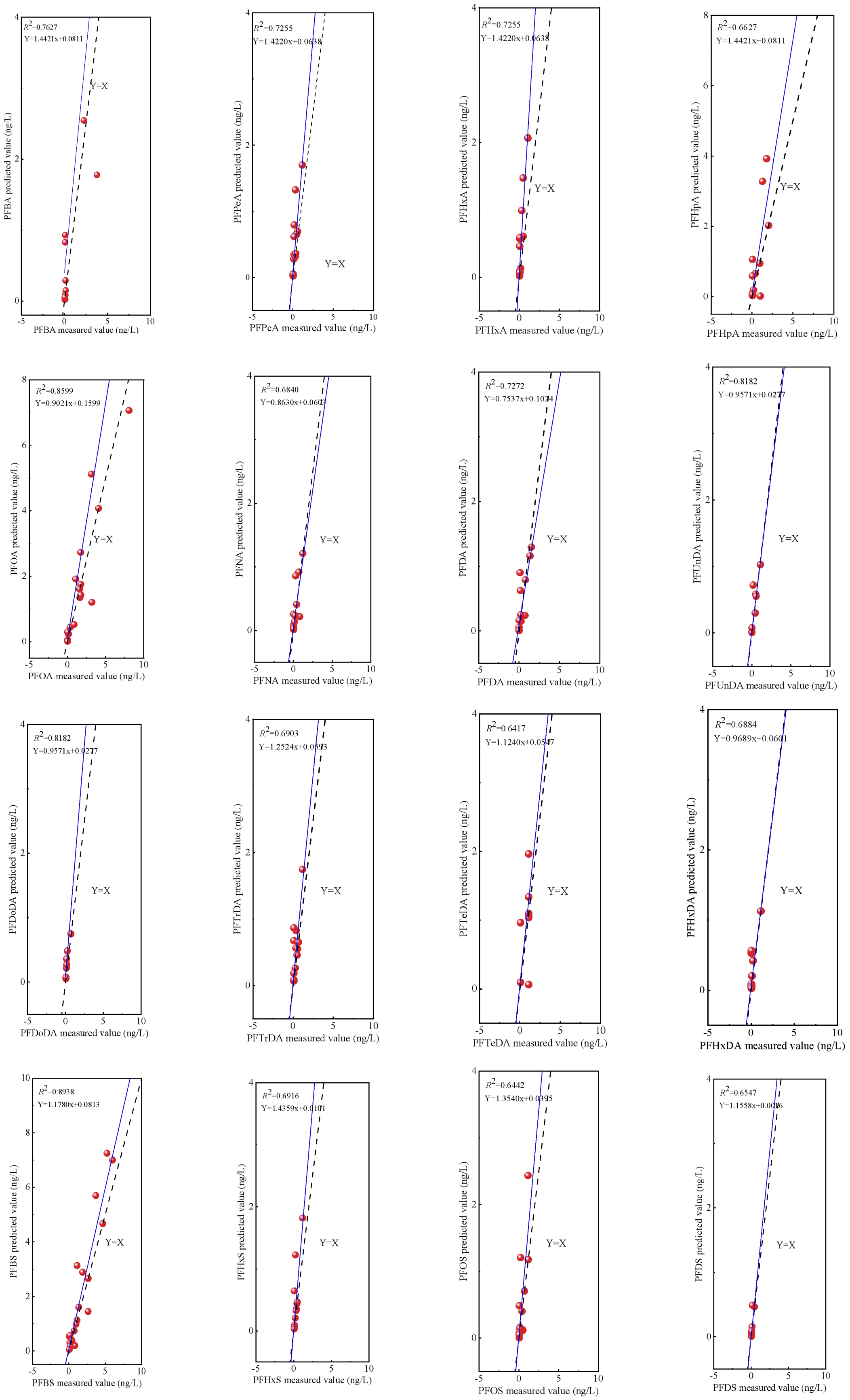

Fig. S7. Correlation between measured value and predicted value of PFASs source contribution rate in lake water body.

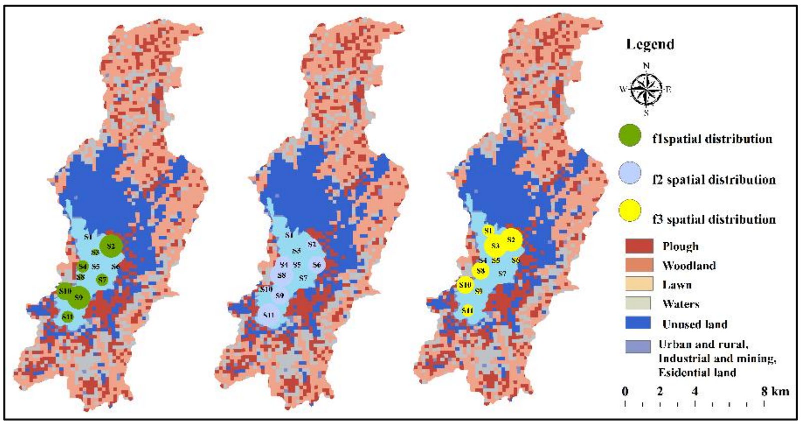

Fig. S8. Spatial distribution of three lake factors and superposition of geographical information.

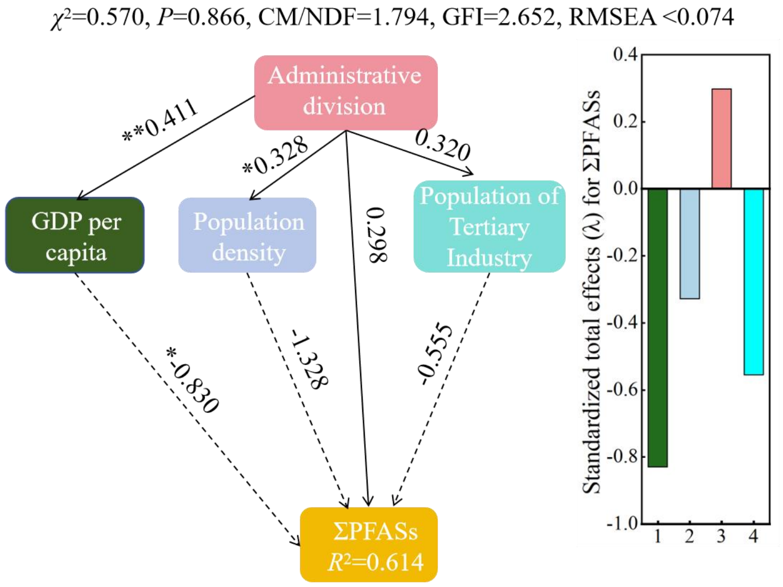

Fig. S9. The structural equation model used to describe the direct and indirect effects of administrative divisions, GDP per capita, population density, and the proportion of tertiary industry on Σ PFASs. (the width of the arrow indicates the strength of the standardized path coefficient(λ). A solid line represents a positive path coefficient, and a dashed line represents a negative path coefficient, The R^2 value represents the proportion of variance explained by each endogenous variable CMIN/DF=the ratio of χ^2 and degrees of freedom, GFI, goodness of fit index, RMSEA, the square root of the approximate error. The significance level of each path is as follows: $*p<0.05$, $**p< 0.01$, $***p <0.001$).
